# Cardiovascular magnetic resonance native T_2_ and T_2_^*^ quantitative values for cardiomyopathies and heart transplantations: a systematic review and meta-analysis

**DOI:** 10.1186/s12968-020-00627-x

**Published:** 2020-05-11

**Authors:** G. J. H. Snel, M. van den Boomen, L. M. Hernandez, C. T. Nguyen, D. E. Sosnovik, B. K. Velthuis, R. H. J. A. Slart, R. J. H. Borra, N. H. J. Prakken

**Affiliations:** 1 Department of Radiology, University Medical Center Groningen, University of Groningen, Hanzeplein 1, 9713 GZ Groningen, The Netherlands; 2 Department of Radiology, Athinoula A. Martinos Center for Biomedical Imaging, Massachusetts General Hospital, Harvard Medical School, 149 13th Street, Charlestown, MA 02129 USA; 3 Cardiovascular Research Center, Massachusetts General Hospital, Harvard Medical School, 149 13th Street, Charlestown, MA 02129 USA; 4 Division of Health Sciences and Technology, Harvard-MIT, 7 Massachusetts Avenue, Cambridge, MA 02139 USA; 5 Department of Radiology, University Medical Center Utrecht, Heidelberglaan 100, 3584 CX Utrecht, The Netherlands; 6 Department of Nuclear Medicine and Molecular Imaging, University Medical Center Groningen, University of Groningen, Hanzeplein 1, 9713 GZ Groningen, The Netherlands; 7 Department of Biomedical Photonic Imaging, University of Twente, Dienstweg 1, 7522 ND Enschede, The Netherlands

**Keywords:** Cardiovascular magnetic resonance imaging, Quantitative values, Cardiomyopathy, Tissue characterization, Myocardium, Edema, Iron, Meta-analysis

## Abstract

**Background:**

The clinical application of cardiovascular magnetic resonance (CMR) T_2_ and T_2_^*^ mapping is currently limited as ranges for healthy and cardiac diseases are poorly defined. In this meta-analysis we aimed to determine the weighted mean of T_2_ and T_2_^*^ mapping values in patients with myocardial infarction (MI), heart transplantation, non-ischemic cardiomyopathies (NICM) and hypertension, and the standardized mean difference (SMD) of each population with healthy controls. Additionally, the variation of mapping outcomes between studies was investigated.

**Methods:**

The PRISMA guidelines were followed after literature searches on PubMed and Embase. Studies reporting CMR T_2_ or T_2_^*^ values measured in patients were included. The SMD was calculated using a random effects model and a meta-regression analysis was performed for populations with sufficient published data.

**Results:**

One hundred fifty-four studies, including 13,804 patient and 4392 control measurements, were included. T_2_ values were higher in patients with MI, heart transplantation, sarcoidosis, systemic lupus erythematosus, amyloidosis, hypertrophic cardiomyopathy (HCM), dilated cardiomyopathy (DCM) and myocarditis (SMD of 2.17, 1.05, 0.87, 1.39, 1.62, 1.95, 1.90 and 1.33, respectively, *P* <  0.01) compared with controls. T_2_ values in iron overload patients (SMD = − 0.54, *P* = 0.30) and Anderson-Fabry disease patients (SMD = 0.52, *P* = 0.17) did both not differ from controls. T_2_^*^ values were lower in patients with MI and iron overload (SMD of − 1.99 and − 2.39, respectively, *P* <  0.01) compared with controls. T_2_^*^ values in HCM patients (SMD = − 0.61, *P* = 0.22), DCM patients (SMD = − 0.54, *P* = 0.06) and hypertension patients (SMD = − 1.46, *P* = 0.10) did not differ from controls. Multiple CMR acquisition and patient demographic factors were assessed as significant covariates, thereby influencing the mapping outcomes and causing variation between studies.

**Conclusions:**

The clinical utility of T_2_ and T_2_^*^ mapping to distinguish affected myocardium in patients with cardiomyopathies or heart transplantation from healthy myocardium seemed to be confirmed based on this meta-analysis. Nevertheless, variation of mapping values between studies complicates comparison with external values and therefore require local healthy reference values to clinically interpret quantitative values. Furthermore, disease differentiation seems limited, since changes in T_2_ and T_2_^*^ values of most cardiomyopathies are similar.

## Background

Ventricular dysfunction in ischemic cardiomyopathies is triggered by impaired coronary blood supply to the myocardium [1]. In non-ischemic cardiomyopathy (NICM) many factors contribute to heart failure (HF) including hypertrophic cardiomyopathy (HCM), dilated cardiomyopathy (DCM) and restrictive cardiomyopathy [2, 3]. The prevalence of HF has been rising since the year 2000 and is shown to be related to the current lifestyle in Western Society [4, 5], with increasing populations with high cardiovascular risk (obesity, hypertension and type 2 diabetes mellitus (T2DM)) [6].

Early diagnosis of cardiomyopathy is important to initiate appropriate treatment [7, 8]. Physical examination and medical history are used to assess the probability of HF, however these assessments are non-specific in early diagnosis and therefore require additional tests [8, 9]. Electrocardiography (ECG) is also used in the first assessment of HF, and although an abnormal ECG increases the probability of HF, it has low specificity and provides little information to distinguish between cardiac diseases [8]. Transthoracic echocardiography was suggested as primary imaging test in the diagnostic pathway of HF because of its wide availability and low costs, and its cardiac function assessment enables fast decision making [8, 10], it however has limitations in distinguishing between underlying diseases [11]. Cardiovascular magnetic resonance (CMR) is the golden standard to detect cardiac remodelling by assessing the global cardiac function, it allows for regional function assessment with strain analysis and furthermore enables the assessment of myocardial fibrosis with late gadolinium enhancement (LGE) [8, 12–14], whereas computed tomography is recommended to either exclude or to diagnose coronary artery disease [8]. Nevertheless, early myocardial structural changes are often qualitatively indistinguishable, and difficult to differentiate from overlapping findings in patients with high cardiovascular risk such as obesity, hypertension and T2DM [15–18]. Consequently, misinterpretation of cardiac remodeling in these high cardiovascular risk groups may result in incorrect diagnosis and mistreatment. The changes occurring in cardiomyopathies, however, may affect myocardial tissue properties, which can be measured quantitatively by T_1_, T_2_ and T_2_^*^ mapping as part of the CMR exam [19]. In line with this, the European Society of Cardiology recently described a shifting standards from the assessment of LGE towards the use of T_1_ and T_2_ mapping in their latest position statement [20]. The clinical utility of T_1_ mapping has already been acknowledged and included in some guidelines [8, 13, 21, 22]. In addition, other guidelines also advocate to include T_2_ and T_2_^*^ mapping instead of T_2_-weighted imaging [20, 22–24].

The Society for Cardiovascular Magnetic Resonance (SCMR) released clinical recommendations about parametric imaging in CMR [22]. T_2_ mapping values vary due to different water concentrations in the myocardium and therefore T_2_ mapping could be useful in infiltrative cardiomyopathies, such as iron overload and Anderson-Fabry disease, and in myocardial injury diseases featuring edema, necrosis, and hemorrhage formation [22, 25, 26]. Furthermore, T_2_ could contribute in the diagnosis of heart transplant rejections as edema correlates with acute heart transplant rejection [22, 27]. In addition to T_2_, T_2_^*^ mapping values mainly depend on magnetic field inhomogeneities and are therefore clinically useful in iron related diseases, and also enable assessment of hemorrhage formation [22, 28, 29].

Reference values of T_2_ and T_2_^*^ mapping in healthy subjects have been investigated in multiple studies [30–33]. The heterogeneity of the data caused by different field strengths, imaging techniques and settings underlines the need for local reference values [22, 33]. The objective of this study was to perform a meta-analysis to determine the weighted mean of myocardial T_2_ and T_2_^*^ mapping values in the HF-related cardiomyopathies and heart transplantations, and standardized mean differences (SMD) with healthy controls. Knowledge of these ranges can help determine the clinically applicability of quantitative techniques. Furthermore, we aim to investigate the presumed heterogeneity of studies leading to variation in mapping outcomes, to emphasize the importance of mapping standardization.

## Materials and methods

### Search strategy

The study was performed according to the Preferred Reporting Items for Systematic Reviews and Meta-Analyses (PRISMA) statement [34] and the Cochrane Handbook for Systematic Review [35]. Three independent investigators (GS, MvdB and LH) systematically searched for eligible studies published between January 2011 and September 2019 in PubMed/MEDLINE and Embase applying CMR T_2_ or T_2_^*^ mapping in humans. The search contained terms related to T_2_ or T_2_^*^ mapping and cardiac diseases (full search terms are listed in Supplementary Data 1).

In this meta-analysis we accepted published results from randomized control trials, cohort studies and observational studies in peer-reviewed journals if they included adults aged 18 years and older with NICM or ischemic cardiomyopathy, heart transplant patients or adults with increased cardiovascular risk, and reported CMR derived T_2_ and/or T_2_^*^ mapping values acquired at 1.5 T or 3 T. Studies were excluded if the article was not available in English or in full text.

### Study selection

Titles and abstracts proposed by the databases were assessed for eligibility by one author and checked by a second author (GS, MvdB and LH). After consensus between these investigators, the full-text reports of these eligible studies were independently assessed by two investigators for final inclusion. The study quality was systematically evaluated with the Newcastle-Ottawa quality assessment scale (NOS) [36]. This scale evaluated the study quality on three domains: selection and definition of included populations (0–4 points); comparability of the controls (0–2 points); and ascertainment of the outcome (0–3 points).

### Data collection

Data were extracted from the included studies by one author and checked by a second author (GS, MvdB and LH). Relevant data regarding patient characteristics, such as; study population, age, gender, body mass index, T_2_ and T_2_^*^ values, as well as CMR imaging acquisition related information, such as; field strength, vendor, sequence and sequence parameters were extracted. Data were reported as mean ± standard deviation (SD) and data reported as median with interquartile or full range were converted using the methodology of Hozo et al. [37]. Healthy control data were extracted if available.

### Data analysis

The included data were divided into two groups of reported T_2_ and T_2_^*^ values per disease and combined into a random effects model to determine the SMD and the 95% confidence interval (CI). The heterogeneity of the included studies was defined with I^2^ being significant if I^2^ ≥ 50% (*P* <  0.05) by using a χ^2^ test. This heterogeneity was further tested by a meta-regression, sensitivity and bias analysis. Available covariates were tested for their association with the myocardial T_2_ and T_2_^*^ values using a backwards elimination model and remaining significant covariates (*P* <  0.05) were included into a mixed effect model of the data. Publication bias was assessed by inspection of the funnel plots with the Egger regression asymmetry test and a sensitivity analysis was performed by omitting each study sequentially and recalculating the model. A meta-analysis was performed in each population with at least 10 published studies, as stated by the PRISMA guideline [34]. Review Manager (RevMan) v. 5.3 (Cochrane Collaboration, Copenhagen, Denmark) was used to determine the random effect models and the package “metaphor” in R v. 3.4.1 (R Foundation for Statistical Computing, Vienna, Austria) was used for the mixed effect models, bias and sensitivity analysis.

## Results

### Literature search

**Fig. 1:**
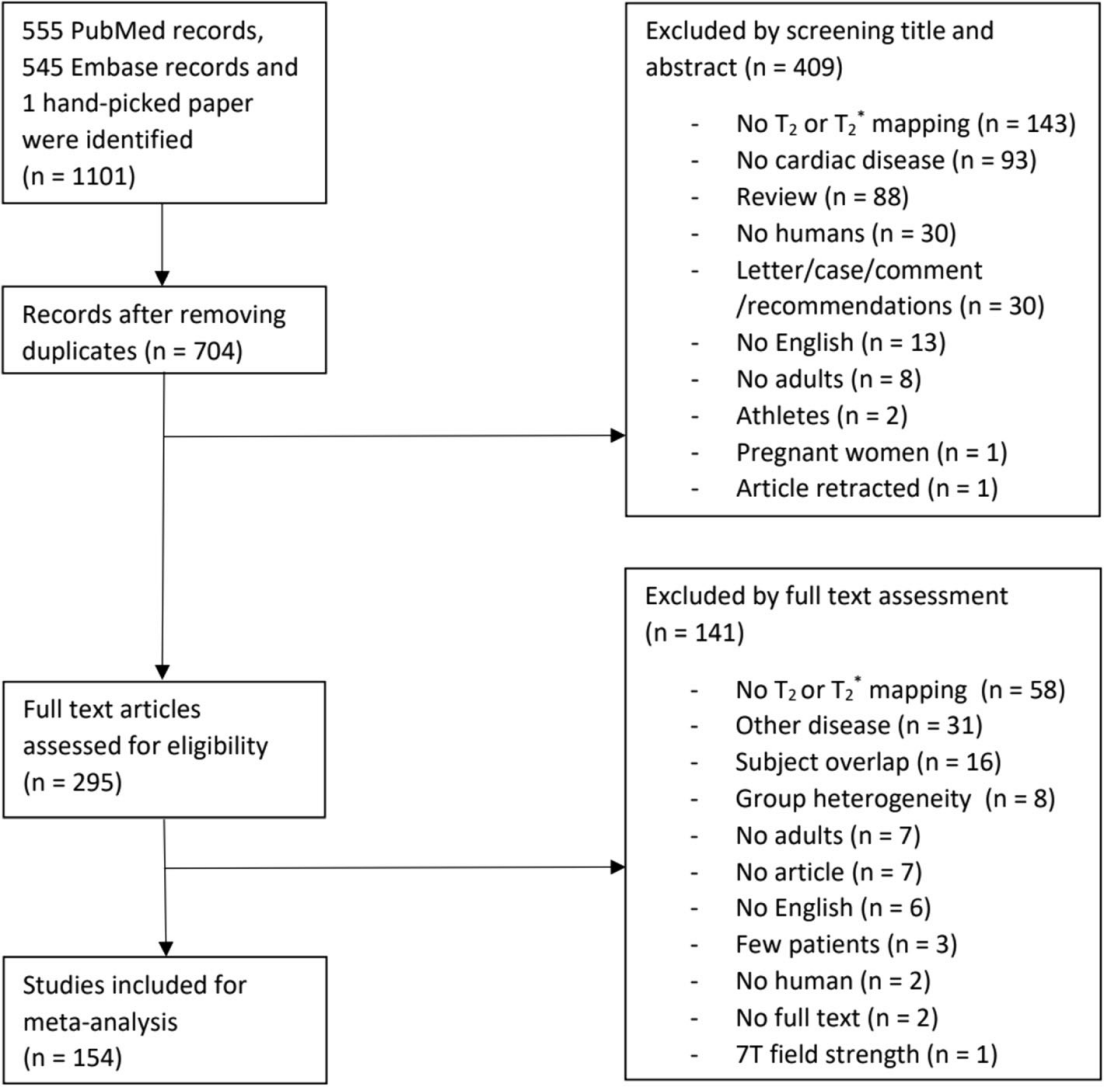
Overview of the study review process according to the PRISMA flow diagram

The search in PubMed and Embase revealed respectively 555 and 545 articles, and one article was manually added [38]. After removal of the duplicates, 704 articles remained for evaluation of title and abstract which resulted in 154 articles included for the final meta-analysis (Table 1). In the final exclusion step based on full text assessment, we excluded studies which presumably included (mostly) the same patient population as other included studies based on authors and method; the study with the least inclusions was excluded. The PRISMA flow diagram with rationale for exclusion is provided in Fig. 1. The number of studies per population was described as total studies (number of studies reporting T_2_ data & number of studies reporting T_2_^*^ data): A total of 31 (22 T_2_ & 13 T_2_^*^) studies were included in the myocardial infarction (MI) population [26, 39–68], 11 (11 T_2_ & 0 T_2_^*^) in heart transplantation [27, 69–78], 70 (5 T_2_ & 70 T_2_^*^) in iron overload [79–148], 2 (2 T_2_ & 0 T_2_^*^) in sarcoidosis [149, 150], 4 (4 T_2_ & 0 T_2_^*^) in systemic lupus erythematosus (SLE) [151–154], 2 (2 T_2_ & 0 T_2_^*^) in amyloidosis [155, 156], 2 (2 T_2_ & 0 T_2_^*^) in Anderson-Fabry disease [157, 158], 4 (2 T_2_ & 2 T_2_^*^) in HCM [159–162], 9 (7 T_2_ & 2 T_2_^*^) in DCM [160, 163–170], 19 (19 T_2_ & 0 T_2_^*^) in myocarditis [25, 38, 171–187] and 1 (0 T_2_ & 1 T_2_^*^) in hypertension [188] (Table 1). The absolute T_2_ and T_2_^*^ values are dependent on field strength [189, 190], therefore the average mapping values were noted separately for 1.5 T and 3 T, and it was also used as covariate in the meta-regression analysis. T_2_ and T_2_^*^ mapping obtained in control subjects were recorded as values from healthy subjects, unless the control population was explicitly defined otherwise in the “population” column of Table 1.

**Table 1:** Characteristics of the included studies in the meta-analysis

First author, year	Disease (n)/ Control (n)	T_2_/T_2_^*^ (ms) Disease	T_2_/T_2_^*^ (ms) Control	*P* value	ROI placement	Seq.	Qual.	Population
**Myocardial Infarction (T**_2_^*^) 1.5 T Philips								
Durighel 2017 [39]	H+: 30	33.8 ± 14.1^a^	45.0 ± 9.4^c^	0.16^bc^	1 SAx at infarct	GRE	1,0,2	STEMI patients referred for CMR in 7 days post-PCI. Haemorrhagic hypointense LGE infarct (H+) or non-haemorrhagic infarcts (H-). Remote as control.
	H-: 30/30	54.0 ± 17.9^b^						
**1.5 T Siemens**								
Bulluck 2016 [40]	CF0: 15	11.3 ± 1.5	32.3 ± 3.9		Segments in 3 SAx		1,0,2	STEMI patients 4d (F0) and 5 m (F1) post-PCI. Hypo-core (C) (T_2_^*^ < 20 ms), infarct (I) 2SD above remote myocardium. Remote as control.
	CF1: 15	15.0 ± 1.5	33.3 ± 3.1					
	IF0: 13	29.7 ± 10.0						
	IF1: 13/28	32.0 ± 5.8						
Bulluck 2017 [41]	26/26	13 ± 3	33 ± 4	< 0.01	Segments		2,0,2	STEMI patients PCI < 2 h, CMR at 4d post-PCI. Hypo-core (T_2_^*^ < 20 ms) measured. Remote as control.
Carberry 2017 [42]	CF0: 203	14.2 ± 3.6	31.5 ± 2.4		3 SAx		2,0,2	STEMI patients 2d (F0) and 6 m (F1) post-PCI. Hypo-core (C) (T_2_^*^ < 20 ms) and infarct zone (Z). Remote as control.
	CF1: 203	16.6 ± 2.1						
	ZF0: 203	32.4 ± 7.6						
	ZF1: 203/203	25.7 ± 4.4						
Carrick 2016 [43]	CF0: 30	17.8 ± 6.0	31.9 ± 2.0		3 SAx		1,0,3	STEMI patients 4–12 h (F0), 3d (F1), 10d (F2) and 7 m (F3) post-PCI. T_2_^*^ in infarct zone (Z) (T_2_ > 2SD remote) and infarct core (C) (center in the infarct zone with mean T_2_/T_2_^*^ value <2SD T_2_/T_2_^*^ periphery). Remote as control.
	CF1: 30	14.1 ± 4.1	32.9 ± 1.9					
	CF2: 30	16.7 ± 5.9	32.6 ± 1.6					
	CF3: 30	18.9 ± 6.2	32.4 ± 2.3					
	ZF0: 30	29.2 ± 5.8						
	ZF1: 30	26.6 ± 4.8						
	ZF2: 30	28.6 ± 3.3						
	ZF3: 30/30	29.2 ± 4.0						
Kali 2013 [44]	H+: 7	15.9 ± 4.5^a^	35.2 ± 2.1^c^	< 0.01^ac^	SAx whole LV	GRE	1,0,2	STEMI patients within 3 days post-PCI. LGE+ infarcts. Hypo-cores on the T_2_^*^-weighted image <2SD reference ROI (H+), otherwise non-haemorrhagic (H-). Remote as control.
	H-: 7/14	37.8 ± 2.5^b^		< 0.05^bc^				
Mohammadzadeh 2018 [45]	I: 20	35.5 ± 3.6^a^	29.4 ± 4.5^c^	< 0.01^ac^	3 SAx & 2 LAx		1,0,2	NSTEMI patients ≥6 months after MI. T_2_^*^ from infarct (I) (LGE+) and peri-infarct (P). Remote as control.
	P: 20/20	30.7 ± 4.9^b^		NS^bc^				
Robbers 2017 [46]	C: 43	26.3 ± 10.7	27.3 ± 6.9		1 SAx at infarct		2,0,2	STEMI patients 4-6d post-PCI. Infarct core (C) (LGE+ based) and border zone (B). Remote as control.
	B: 43/43	30.7 ± 7.7						
Roghi 2015 [47]	H + F0: 7	17			3 SAx at necrotic area	GRE	1,0,1	STEMI patients < 5 days (F0) and 6 m (F1) post-PCI. LGE+ as myocardial haemorrhagic (H+) (dark core at T_2_^*^) or non-haemorrhagic (H-).
	H + F1: 6	18						
	H-F0: 8	31						
	H-F1: 8	31						
Yilmaz 2013 [48]	I: 14	24.0 ± 12.4	32.0 ± 4.9		3 SAx at infarct	GRE	1,0,2	STEMI patients 2–7 days post-PCI. Infarct core (LGE+ with hyperenhanced T_2_ area) and peri-infarct zone (P) (LGE area without hyperenhanced T_2_ area). Remote as control.
	P: 14/14	35.7 ± 10.7						
**1.5 T GE**								
Zia 2012 [49]	F0: 62	32.4^a^	37.4^d^	< 0.01^ad^	3 SAx at infarct	GRE	2,0,2	STEMI patients within 2d (F0), 3w (F1) and 6 m (F2) post-PCI. LGE+ infarct. Remote as control.
	F1: 62	37.7^b^	38.4^e^	NS^be^				
	F2: 62/62	37.3^c^	38.2^f^	NS^cf^				
**Myocardial Infarction (T**_2_^*^) 3 T Philips								
Chen 2019 [50]	F0: 22	22.0 ± 3.1	31.2 ± 1.6		3 SAx	TFE	2,0,2	STEMI patients 1d (F0), 3d (F1), 7d (F2) and 30d (F3) post-PCI. Infarct values (LGE+ based). Remote as control.
	F1: 22	23.9 ± 3.3	30.0 ± 0.7					
	F2: 22	22.1 ± 4.0	30.4 ± 0.8					
	F3: 22/22	21.5 ± 2.8	30.3 ± 0.7					
Zaman 2014 [51]	6/15	16.1 ± 7.6	24.2 ± 6.7		Stack of SAx	GRE	2,0,2	STEMI patients 2d post-PCI. Intramyocardial haemorrhage (hypo-core on LGE+).
**Myocardial Infarction (T**_**2**_**) 1.5 T Philips**								
Nakamori 2019 [52]	14	45			Mean 16 AHA		1,0,1	Patients with coronary artery disease.
Tahir 2017 [53]	F0: 67	84 ± 10	55 ± 3		Mid-SAx	TSE	2,0,3	Acute MI patients 8d (F0), 7w (F1), 3 m (F2) and 6 m (F3) post-PCI. Infarct (LGE+ area without hypo-intense area). Remote as control.
	F1: 50	68 ± 9						
	F2: 44	61 ± 7						
	F3: 45/67	58 ± 4						
**1.5 T Siemens**								
Bulluck 2016 [40]	F0: 15	49.7 ± 5.7	49.3 ± 2.5		3 SAx		1,0,2	STEMI patients 4d (F0) and 5 m (F1) post-PCI. Hypo-core (T_2_^*^ < 20 ms). Remote of another population as control.
	F1: 15/13	47.3 ± 4.1	46.7 ± 2.5					
Bulluck 2017 [41]	H + C: 26	50 ± 4	51 ± 3		3 SAx		2,0,2	STEMI patients 4d post-PCI. Hypo-core (H+) (T_2_^*^ < 20 ms) and without (H-) in infarct core (C) (LGE+) or salvage (S). Remote as control.
	H + S: 26	66 ± 6	50 ± 3					
	H-C: 13	57 ± 4						
	H-S: 13	66 ± 7						
	H + R: 26							
	H-R: 13							
Carberry 2017 [54]	F0: 283	66.3 ± 6.1^a^	49.7 ± 2.3^c^	< 0.01^ac^	SAx whole LV	T_2_-prep tFISP	1,0,2	STEMI patients 2d (F0) and 6 m (F1) post-PCI. Infarct (SI > 5SD above remote region). Remote as control.
	F1: 283/283	56.8 ± 4.5^b^		< 0.01^bc^				
Carrick 2016 [43]	CF0: 30	55.5 ± 6.9	49.5 ± 2.5		SAx	T_2_-prep tFISP	1,1,3	STEMI patients 4-12 h (F0), 3d (F1), 10d (F2) and 7 m (F3) post-PCI. Infarct zone (I) (T_2_ > 2SD above remote) and infarct core (C) (center infarct with a mean T_2_/T_2_^*^ value >2SD below periphery).
	CF1: 30	51.8 ± 4.6						
	CF2: 30	59.2 ± 3.6						
	IF0: 30	62.8 ± 6.7						
	IF1: 30	61.4 ± 4.1						
	IF2: 30	68.1 ± 3.7						
	IF3: 30/50	54.0 ± 2.8						
Carrick 2016 [55]	171	54 ± 5			SAx whole LV	T_2_-prep tFISP	2,0,2	STEMI patients 2d post-PCI. Infarct core (T_1_ < 2SD of periphery).
Haig 2018 [56]	C: 245	53.9 ± 4.8	49.7 ± 2.1		SAx whole LV	T_2_-prep tFISP	1,0,3	STEMI patients 2d post-PCI. Infarct zone (Z) (T_2_ > 2SD above remote) and core (C) (center infarct with a mean T_2_/T_2_^*^ > 2SD below periphery). Remote as control.
	Z: 245/245	62.9 ± 5.1						
Hausenloy 2019 [57]	I: 48	66 ± 6	50 ± 3		1 SAx		1,0,1	STEMI patients 4d post-PCI. Infarct (I) (LGE area+) and salvaged (S) (LGE- epicardial to infarcted). Remote as control.
	S: 48/ 48	64 ± 6						
Krumm 2016 [58]	22/10	83 ± 23	50 ± 6		3 SAx	FSE	1,0,2	STEMI patients 1-5d post-PCI. Infarct (LGE+ based).
McAlindon 2014 [59]	40/40	71	54		3 SAx	T_2_-prep SSFP	2,0,2	STEMI patients 1-4d post-PCI. Myocardial edema (area with abnormal SI). Remote as control.
Masci 2018 [60]	C: 163	47.3 ± 3.8	45.5 ± 3.0		1 SAx at infarct	T_2_-prep SSFP	1,0,2	STEMI patients 2.7 days (median) post-PCI. Infarct (I) (LGE+ SI > 5SD remote) and infarct core (C) (hypo-core in LGE+). Remote as control.
	I: 163/163	62.8 ± 6.4						
Park 2013 [61]	20/7	67.9 ± 9.3	52.4 ± 3.0		SAx whole LV	T_2_-prep SSFP	2,0,2	Acute MI patients scanned < 7 days post-PCI. Infarct (LGE+ SI > 5SD remote).
Tessa 2018 [62]	47/47	69 ± 9	51.9 ± 2.9	< 0.01	3 SAx & 2 LAx	T_2_-prep tFISP	1,0,2	Acute NSTEMI patients before coronary angiography. Infarct (LGE > 2SD remote). Remote as control.
Verhaert 2014 [26]	27/21	69 ± 6	55.5 ± 2.3		3 SAx & 2 LAx	T_2_-prep SSFP	2,0,2	STEMI and NSTEMI patients 2.1d (mean) after hospital admission. Infarct (LGE+).
White 2014 [63]	40/40	73.1 ± 6.1	50.1 ± 2.0		SAx whole LV	T_2_-prep SSFP	2,0,2	STEMI patients 3-6d post-PCI. Infarct (LGE+). Remote as control.
**1.5 T GE**								
Zia 2012 [49]	F0: 62	56.7^a^	43.4^d^	< 0.01^ad^	5 SAx at infarct	T_2_-prep SI	2,0,2	STEMI patients 2d (F0), 3w (F1) and 6 m (F2) post-PCI. LGE+ segments. Remote as control.
	F1: 62	51.8^b^	39.5^e^	< 0.01^be^				
	F2: 62/62	39.8^c^	39.5^f^	NS^cf^				
**Myocardial Infarction (T**_**2**_**) 3 T Philips**								
An 2018 [64]	F0: 20	66.7 ± 4.7^a^	53.6 ± 5.3^e^	< 0.05^ae^	3 SAx	GraSE	2,0,2	STEMI patients 1d (F0), 3d (F1), 7d (F2) and 30d (F3) post-PCI at infarct.
	F1: 20	73.6 ± 4.4^b^		< 0.05^be^				
	F2: 20	68.4 ± 4.2^c^		< 0.05^ce^				
	F3: 20/12	65.0 ± 5.4^d^		< 0.05^de^				
Zaman 2014 [51]	6/15	81 ± 52	39.1 ± 6.0		SAx whole LV	SE	2,0,2	STEMI patients 2d post-PCI. Edematous myocardium (T_2W_ > 2SD above SI remote).
**3 T Siemens**								
Bulluck 2016 [65]	21	58.4 ± 7.9			SAx whole LV		1,0,1	STEMI patients 4-6d post-PCI. Segments ≥50% transmural LGE.
Fischer 2018 [66]	26/10	40.7 ± 4.0	38.4 ± 1.7		Basal and mid-SAx	GRE	3,0,2	Patients with an untreated vascular territory of > 50% diameter stenosis. Territories affected by this stenosis.
Layland 2017 [67]	73/73	57 ± 5	45 ± 3	< 0.01	3 SAx	T_2_-prep tFISP	1,0,2	NSTEMI patients 6.5d (mean) after invasive management. Infarct (LGE+ > 2SD remote). Remote as control.
Van Heeswijk 2012 [68]	11/10	61.2 ± 10.1	38.5 ± 4.5		Mid-SAx	T_2_-prep GRE	1,0,2	STEMI patients in subacute phase post-PCI. Infarct (area on LGE+ > 3SD remote).
**Heart Transplantation (T**_**2**_**) 1.5 T Siemens**								
Butler 2015 [69]	B-: 58	57 ± 6			Septal SAx	FSE	2,0,1	Heart transplant patients classified on EMB grades between negative (B-) and positive (B+) biopsy.
	B+: 15	63 ± 6						
Dolan 2018 [70]	61/14	50.5 ± 3.4	45.2 ± 2.3	< 0.01	Mean 16 AHA	T_2_-prep SSFP	1,1,2	Heart transplant patients for regular follow-up.
Dolan 2019 [71]	R-: 36	49.2 ± 4.0	45.2 ± 2.3		Mean 16 AHA	T_2_-prep SSFP	1,2,2	Heart transplant patients classified between without (R-) and with acute cardiac allograft rejection (R+).
	R+: 23/14	52.4 ± 4.7						
Markl 2013 [72]	0R: 8	53.4 ± 1.8	52.2 ± 1.8		Mean 16 AHA	T_2_-prep SSFP	1,1,2	Heart transplant patients with no rejection (0R) or mild rejection (1R).
	1R: 2/14	56.1 ± 1.5						
Miller 2014 [73]	0&1R: 22	57.0 ± 3.2^a^	54.1 ± 2.0^c^	< 0.01^ac^	Mean mid-SAx	T_2_-prep SSFP	3,2,2	Heart transplant patients classified based on biopsy: 0&1R = absence of rejection and 2R = presence of rejection.
	2R: 22/10	58.8 ± 3.5^b^		< 0.01^bc^				
Miller 2019 [74]	R-: 26	47.0 ± 1.7			Mid-SAx excluding LGE+	T_2_-prep SSFP	2,0,1	Heart transplant patients classified as no rejection (R-), biopsy negative rejection (BNR; allograft rejection with normal biopsy), acute cellular rejection (ACR; 2R or 3R cellular rejection, or treated 1R) and anti-body mediated rejection (AMR; biopsy with grade 2 or 1 with clinically impression of AMR).
	BNR: 12	51.8 ± 2.4						
	ACR: 5	53.4 ± 3.1						
	AMR: 3	55.2 ± 2.8						
Usman 2012 [27]	0R: 46	52.5 ± 2.2	52.2 ± 3.4		Mean 16 AHA	T_2_-prep SSFP	1,0,2	Heart transplant patients classified based on EMB transplant rejection grades: 0R = no rejection, 1R = mild rejection, 2R = moderate rejection and 3R = severe rejection.
	1R: 17	53.1 ± 3.3						
	2R: 3	59.6 ± 3.1						
	3R: 1/14	60.3						
Vermes 2018 [75]	B-: 24	51.8 ± 2.8^a^	51.0 ± 3.1^c^	NS^ac^	Mean 16 AHA	T_2_-prep SSFP	1,0,2	Heart transplant patients classified based on EMB transplant rejection grades between negative (B-) and positive (B+).
	B+: 7/34	56.5 ± 5.2^b^		< 0.05^bc^				
Yuan 2018 [76]	58/20	47.7 ± 2.8	44.5 ± 1.6	< 0.01	Mean basal and mid-SAx	T_2_-prep SSFP	3,2,2	Heart transplant patients without EMB proven rejection.
**1.5 T GE**								
Bonnemains 2013 [77]	0R: 14	55.0 ± 2.3			Septal mid-SAx	FSE	2,0,1	Heart transplant patients classified based on EMB transplant rejection grades: 0R = no rejection, 1R = mild rejection and 2&3R = moderate & severe rejection.
	1R: 42	64.1 ± 11.0						
	2&3R: 19	72.1 ± 9.0						
Odille 2015 [78]	9	62.2 ± 11.2			Mean mid-SAx	FSE	1,0,1	Heart transplant patients without biopsy.
**Iron Overload (T**_2_^*^) 1.5 T Philips								
Desai 2015 [79]	38/13	41.6 ± 13.4	38.4 ± 14.4	0.91	Septal mid-SAx		1,2,2	Clinically stable sickle cell disease subjects.
Fragasso 2011 [80]	TM: 99	27 ± 15			Mean septal 3 SAx		2,0,1	Three groups of multi-transfused patients: all TM, all TI patients and 60% of the acquired anemia patients were on chelation therapy.
	TI: 20	30 ± 11						
	AA: 10	33 ± 11						
Kritsaneepaiboon 2017 [81]	42/20	35.7 ± 6.9	36.7 ± 3.0	0.63	Septal mid-SAx	GRE	1,0,2	Iron-overloaded patients suffering from primary or secondary hemochromatosis referred for cardiac siderosis screening or follow up.
Krittayaphong 2017 [82]	200	37.8 ± 7.0			Septal mid-SAx	GRE	1,0,1	Thalassemia patients treated with blood transfusions (85%) and chelation therapy (76%).
Portillo 2013 [83]	16	28.7 ± 5.7			Mean septal 3 SAx	GRE	1,0,1	Polytransfused patients and one anemia patient.
Saiviroonporn 2011 [84]	50	31.4 ± 13.8			Septal mid-SAx	GRE	1,0,1	Regular transfused TM patients on iron chelation therapy.
Seldrum 2011 [85]	19/8	22 ± 11	40 ± 10	< 0.01	Septal mid-SAx	GRE	3,1,2	Chronic anaemia patients on transfusion treatment.
Soltanpour 2018 [86]	60	23.8 ± 12.1				GRE	2,0,1	Regular transfused ß-TM patients receiving chelation therapy.
**1.5 T Siemens**								
Acar 2012 [87]	22	23.7 ± 11.2			Mean mid-SAx	GRE	1,0,1	Regular transfused ß-TM diagnosed patients (every 3–4 weeks) and receiving chronic chelation therapy.
Alam 2016 [88]	104/20	30.0 ± 10.5	32.7 ± 6.4	0.20	Septal mid-SAx		2,0,2	Transfusion dependent anemia patients referred for siderosis screening.
Alp 2014 [89]	38	22.9 ± 13.3					1,0,1	Regular transfused ß-TM patients (≥ 15/year) and receiving chelation therapy.
Azarkeivan 2013 [90]	156	24.6 ± 15.1			Septal mid-SAx	GRE	1,0,1	Regular transfused TM patients and receiving chelation therapy.
Barzin 2012 [91]	33	20.4 ± 12.1			Septal mid-SAx	GRE	1,0,1	TM patients transfused for a least 15 years.
Bayraktaroglu 2011 [92]	47	14.1			Mean septum		1,0,1	Regular transfused TM patients and receiving chelation therapy with cardiac involvement (T_2_^*^ < 20 ms).
Camargo 2016 [93]	7/17	15.4 ± 6.0	28.0 ± 4.0	< 0.01	Septal mid-SAx	GRE	3,0,2	Patients with myocardial iron overload (T_2_^*^ < 20 ms), regardless of chelating therapy status.
Cassinerio 2012 [94]	67	24.5 ± 12.7			Septal mid-SAx	GRE	1,0,1	ß-TM patients treated with iron chelators
Delaporta 2012 [95]	44/143	11.0 ± 5.6	33.5 ± 5.1	< 0.01			1,0,2	ß-TM patients with LVEF < 50%, regularly transfused (2–3 weeks), on chelation therapy and cardiac siderosis (T_2_^*^ < 20 ms). ß-TM patients without cardiac siderosis (T_2_^*^ ≥ 20 ms) as controls.
Di Odoardo 2017 [96]	21/34	12.1 ± 4.7	35.7 ± 9.5	< 0.01	Septal mid-SAx	GRE	2,0,2	ß-TM patients on long-term iron-chelation therapy with cardiac involvement (T_2_^*^ < 20 ms). ß-TM patients without cardiac involvement (T_2_^*^ ≥ 20 ms) as controls.
Djer 2013 [97]	30	24.3 ± 11.2			Mean septum		2,0,1	TM patients with at least 13 years transfusion history and chelation therapy.
Ebrahimpour 2012 [98]	TM: 49	24.9 ± 13.6			Septal mid-SAx	GRE	2,0,1	ß-TM and TI patients on regular transfusion therapy.
	TI: 29	29.7 ± 12.8						
Eghbali 2017 [99]	56	22.9 ± 7.3					1,0,1	TM patients on chelation therapy.
Fahmy 2015 [100]	70	32.1 ± 12.1			Mean septal 3 mid-SAx	GRE	1,0,1	ß-TM and sickle cell anaemia patients on regular transfusion program and iron chelation therapy referred for cardiac/liver siderosis.
Feng 2013 [101]	106	22.3 ± 24.0			Septal mid-SAx	GRE	1,0,1	Regularly transfused TM patients receiving iron chelation therapy.
Fernandes 2011 [102]	60	31.2 ± 10.3			Septal mid-SAx	GRE	2,0,1	TM patients receiving chronic transfusion therapy and iron chelation regimen.
Fernandes 2016 [103]	56	34.7 ± 11.8				GRE	1,0,1	TM, hemochromatosis and sickle cell anemia patients on transfusion therapy.
Garceau 2011 [104]	22/23	11 ± 4	33 ± 8		Mean septal basal and mid-SAx		2,0,2	Chronically transfused ß-TM patients or Diamond-Blackfan anaemia, with cardiac involvement (T_2_^*^ < 20 ms). Patients without cardiac involvement (T_2_^*^ ≥ 20 ms) as controls.
Git 2015 [105]	50	25.3 ± 1.6			Mid-SAx	GRE	1,0,1	Patients (80% TM) referred for iron overload assessment.
Hanneman 2013 [106]	108	24.3 ± 11.5			Mean 16 AHA	GRE	1,0,1	Transfusion dependent anaemia patients receiving iron chelation therapy.
Hanneman 2015 [107]	19/10	24.1 ± 9.2	35.1 ± 5.4	< 0.01	Septal mid-SAx	GRE	3,0,2	TM patients receiving regularly blood transfusions and treatment with iron chelation therapy.
Junqueira 2013 [108]	30	37.6 ± 7.1			Septal mid-SAx		2,0,1	Sickle cell disease patients referred of whom 27 receiving transfusions.
Kayrak 2012 [109]	22	21.7 ± 9.0			Mid-SAx	GRE	1,0,1	ß-TM patients regularly transfused (every 3–4 weeks) and receiving chronic chelation therapy.
Kirk 2011 [110]	45	23.7 ± 16.9			Septal mid-SAx		1,0,1	ß-TM patients receiving chelation therapy (except 1).
Kucukseymen 2017 [111]	56	28.3 ± 13.7					1,0,1	TM patients transfused every 3–4 weeks.
Li 2017 [112]	24	32.7 ± 16.7			Septal mid-SAx		1,0,1	Transfusion-dependent ß-TM patients.
Liguori 2015 [113]	41/145	11.0 ± 8.1	32.1 ± 5.7		Septal mid-SAx	GRE	1,0,2	Regular transfused TM patients under iron chelation therapy and occasionally transfused TI patients with cardiac involvement (T_2_^*^ < 20 ms). Patients without cardiac involvement (T_2_^*^ ≥ 20 ms) as controls.
Mehrzad 2016 [114]	S: 11	8.1 ± 1.4	26.9 ± 6.4		Mid-SAx		1,0,2	Transfusion dependent ß-TM patients with LVEF > 50% classified between severe (S) (T_2_^*^ < 10 ms) and moderate (M) (10 ms < T_2_^*^ < 20 ms) cardiac iron overload. Patients without cardiac involvement (T_2_^*^ > 20 ms) as controls.
	M: 23/16	14.1 ± 2.6						
Ozbek 2011 [115]	21	21.7 ± 9.3			Mid-SAx	GRE	1,0,1	Regularly transfused (every 3–4 weeks) TM patients receiving chronic chelation treatment.
Quatre 2014 [116]	48	21.2 ± 10.1			Septum	GRE	2,0,1	Multi transfused TM and TI patients. 45/48 were receiving iron chelation therapy.
Roghi 2015 [117]	43	31 ± 15			Septal mid-SAx	GRE	2,0,1	TM patients
Sado 2015 [118]	88/67	27 ± 11	31 ± 4	< 0.01	Septal mid-sax		3,0,2	Suspected iron overload patients with several underlying diseases.
Sakuta 2010 [119]	19	45.1 ± 22.4			Mid-SAx		1,0,1	Transfusion-dependent patients without consecutive oral chelation therapy.
Torlasco 2018 [120]	138	38.5 ± 14.1			Septal mid-SAx		1,0,1	TM patients.
**1.5 T GE**								
Chen 2014 [121]	50	26.1 ± 23.0			Mean septum		2,0,2	TM patients transfused every 2–4 weeks.
de Assis 2011 [122]	115	25.0 ± 14.2			Mean septum	GRE	1,0,1	Chronically transfused TM and TI patients.
de Assis 2011 [123]	115	14.3 ± 2.4			Mean septum	GRE	2,0,1	ß-TM patients transfused every 2–3 weeks.
de Sanctis 2016 [124]	6/8	17.5 ± 6.9	36.5 ± 12.5	< 0.01			3,2,2	Regular transfused TM patients and receiving chelation therapy with acquired hypogonadotropic hypogonadism (AHH). TM patients without AHH and T_2_^*^ > 20 ms as controls.
Marsella 2011 [125]	149	19.3 ± 11.9			Mean 16 AHA		2,0,1	TM patients with transfusions every 2–4 week and iron chelation with heart dysfunction.
Mavrogeni 2013 [126]	30	37.2			Septal mid-SAx	GRE	1,0,1	Transfused TM patients (every 2–3 weeks) and receiving iron chelation therapy.
Meloni 2012 [127]	38	30.8 ± 11.3			Mean 16 AHA	GRE	1,0,2	Transfusion dependent patients enrolled in the myocardial iron overload in thalassemia network.
Meloni 2014 [128]	138/329	8.9 ± 2.8	38.7 ± 4.5		Mean 16 AHA	GRE	2,0,2	Regularly transfused TM patients with homogeneous myocardial iron overload (all segments T_2_^*^ < 20 ms). TM without (all segments T_2_^*^ ≥ 20 ms) as controls.
Pepe 2018 [129]	481	27.4 ± 12.4			Mean 16 AHA	GRE	2,0,1	TM patients.
Pistoia 2019 [130]	HE: 279	35.0 ± 14.0			Mean 16 AHA	GRE	2,0,1	TM patients classified: heterozygotes ß^+^/ ß^0^, homozygote ß^+^ and homozygote ß^0^
	ß^+^: 154	32.0 ± 21.0						
	ß^0^: 238	28.5 ± 23.5						
Pizzino 2018 [131]	28	39.0 ± 9.4			Mean 16 AHA		2,0,1	Regularly transfused TM patients receiving chelation therapy.
Positano 2015 [132]	S: 20	7.0 ± 2.4	34.3 ± 5.0		Mean 16 AHA		1,0,2	TM patients were classified as severe (S) (T_2_^*^ < 10 ms) or mild-moderate (M) (10 ms ≤ T_2_^*^ ≤ 20 ms) cardiac involvement. TM patients without cardiac involvement (T_2_^*^ > 20 ms) as controls .
	M: 20/20	15.8 ± 2.4						
Russo 2011 [133]	40/40	29 ± 15	55 ± 13	< 0.05		GRE	4,2,2	ß-TM patients receiving regular blood transfusions (2–4 week) and iron chelation therapy.
Wijarnpreecha 2015 [134]	99	44.3 ± 6.8			Mid-SAx	GRE	1,0,1	Non-transfusion dependent thalassemia and receiving < 7 transfusions per year.
**1.5 T Vendor unknown**								
Barbero 2016 [135]	46	37.7 ± 11.0					2,0,1	Regular transfused ß-TM patients receiving iron chelation and follow-up after 4 years.
		41.0 ± 15.7						
Bayar 2015 [136]	43/60	13 ± 3	33 ± 10	< 0.01			1,0,2	TM patients on regular blood transfusion and iron chelators with cardiac involvement (T_2_^*^ < 20 ms). TM patients without cardiac involvement (T_2_^*^ ≥ 20 ms) as control.
Du 2017 [137]	92	31.9 ± 14.1					1,0,1	Aplastic anaemia patients and myelodysplastic syndrome patients with cardiac iron overload, with multiple transfusions.
Ferro 2017 [138]	45	32.5 ± 12.5					1,0,1	Transfused ß-TM patients.
Karakus 2017 [139]	30/72	14.5 ± 2.1	37.3 ± 12	< 0.01			1,0,2	ß-TM and TI patients with transfusion and chelation therapy with cardiac or hepatic iron overload (T_2_^*^ < 20 ms). Patients without cardiac or hepatic iron overload as controls.
Karami 2017 [140]	6	16.7 ± 15.4					1,0,1	ß-TM patients with regular transfusion and chelation therapy and high serum ferritin levels or severe iron overload
Monte 2012 [141]	27	27.2 ± 12.3					1,0,1	TM patients with LVEF > 55% with transfusions every 3 weeks and iron chelation therapy.
Parsaee 2017 [142]	55	23.5 ± 9.8					1,0,2	TM patients receiving blood transfusions and undergoing iron chelation therapy.
Pennell 2014 [143]	103	11.4 ± 3.5					2,0,2	ß-TM patients with myocardial T_2_^*^ between 6 and 20 ms, LVEF > 55% and transfusion history.
Piga 2013 [144]	924	30.1 ± 14.6					2,0,1	TM patients.
Porter 2013 [145]	20	7.7 ± 4.6				GRE	2,0,1	Transfusion-dependent TM patients with decreased LVEF and cardiac involvement (T_2_^*^ ≤ 20 ms).
Vlachaki 2015 [146]	23	32.8 ± 10.9			Septal mid-SAx		2,0,1	Regularly ß-TM patients excluding patients with decreased LVEF ≤60% or increased cardiac iron overload (T_2_^*^ < 8 ms).
Yuksel 2016 [147]	57	27.6 ± 13.9			Septal mid-SAx	GRE	1,0,1	ß-TM patients.
**Iron overload (T**_2_^*^) 3 T Philips								
Kritsaneepaiboon 2017 [81]	42/20	21.7 ± 6.1	23.7 ± 2.4	0.07	Septal mid-SAx	GRE	1,0,2^1^	Iron-overloaded patients suffering from primary or secondary hemochromatosis referred for cardiac siderosis screening or follow up.
**3 T Siemens**								
Alam 2016 [88]	104/20	18.3 ± 9.0	21.0 ± 4.8	0.14	Septal mid-SAx		2,0,2	Transfusion dependent anemia patients referred for siderosis screening.
Gu 2013 [148]	D+: 33	19.9 ± 2.2			Septum	GRE	2,0,1	Myelodysplastic syndrome patients defined as transfusion dependent (D+) or independent (D-).
	D-: 40	27.0 ± 2.1						
**3 T GE**								
Meloni 2012 [127]	38	27.6 ± 11.8			Mean 16 AHA		1,0,2	Transfusion dependent patients enrolled in the myocardial iron overload in thalassemia network.
**Iron Overload (T**_**2**_**) 1.5 T Philips**								
Kritsaneepaiboon 2017 [81]	42/20	60.3 ± 6.9	58.3 ± 3.2	0.23	Septal mid-SAx	TSE	1,0,2	Iron-overloaded patients suffering from primary or secondary hemochromatosis referred for cardiac siderosis screening or follow up.
Krittayaphong 2017 [82]	200	58.9 ± 7.3			Septal mid-SAx	SE	1,0,1	Thalassemia patients referred for CMR.
**1.5 T Siemens**								
Feng 2013 [101]	106	48.9 ± 22.2			Septal mid-SAx	TSE	1,0,1	Regularly transfused TM patients receiving iron chelation therapy.
**Iron overload (T**_**2**_**) 3 T Philips**								
Kritsaneepaiboon 2017 [81]	42/20	55.7 ± 6.1	58.0 ± 7.2	0.20	Septal mid-SAx	SE	1,0,2	Iron-overloaded patients suffering from primary or secondary hemochromatosis referred for cardiac siderosis screening or follow up.
**3 T Siemens**								
Camargo 2016 [93]	7/17	37.9 ± 6.0	45.0 ± 2.0	< 0.05	Septal mid-SAx	T_2_-prep SSFP	3,0,2	Patients with myocardial iron overload (T_2_^*^ < 20 ms) regardless of chelating therapy.
**Sarcoidosis (T**_**2**_**) 1.5 T Siemens**								
Greulich 2016 [149]	61/26	52.3 ± 3.8	49.0 ± 1.6	< 0.01	Mean mid-SAx	T_2_-prep SSFP	2,2,2	Clinically diagnosed or biopsy proven systemic sarcoidosis patients.
**Sarcoidosis (T**_**2**_**) 3 T Philips**								
Puntmann 2017 [150]	53/36	54.0 ± 12.2	45.0 ± 10.8	< 0.01	Septal mid-SAx	GraSE	3,0,2	Biopsy proven extra cardiac systemic sarcoidosis patients.
**Systemic lupus erythematosus (T**_**2**_**) 1.5 T Siemens**								
Mayr 2016 [151]	13/20	51.0 ± 3.3	49.3 ± 2.4	< 0.01	Mid-SAx	T_2_-prep SSFP	3,0,2	SLE patients.
Zhang 2015 [152]	24/12	58.2 ± 5.6	52.8 ± 4.4		Mid-SAx	T_2_-prep SSFP	3,0,2	SLE patients.
**Systemic lupus erythematosus (T**_**2**_**) 3 T Philips**								
Hinojar 2016 [153]	76/46	65 ± 8	45 ± 4	< 0.01	Septal mid-SAx	GraSE	3,2,2	SLE patients with clinical suspected myocarditis.
Winau 2018 [154]	92/78	51 ± 9	44 ± 4	< 0.01	Septal mid-SAx	GraSE	3,2,2	SLE patients without cardiac disease referred for cardiovascular involvement screening.
**Amyloidosis (T**_**2**_**) 1.5 T Siemens**								
Kotecha 2018 [155]	AL1: 35	53.2 ± 3.6	48.9 ± 2.0		Basal to mid-septum of 4CH	T_2_-prep SSFP	3,0,2	Amyloidosis patients categorized in systemic AL (1. Cardiac with transmural LGE; 2. Cardiac with subendocardial LGE; 3. No signs of cardiac involvement (CA) and ATTR (AT) (1. TTR gene carrier; 2. Possible CA; 3. Definite CA).
	AL2: 37	56.3 ± 4.8						
	AL3: 28	56.2 ± 5.4						
	AT1: 11	50.4 ± 3.2						
	AT2: 12	51.5 ± 3.7						
	AT3: 163/30	54.7 ± 4.0						
Ridouani 2018 [156]	AL: 24	63.2 ± 4.7^a^	51.1 ± 3.1^c^	< 0.01^ac^	Mean mid-SAx and 4CH	T_2_-prep SSFP	2,0,2	Amyloidosis patients with cardiac involvement classified as AL or ATTR (AT).
	AT: 20/40	56.2 ± 3.1^b^		< 0.01^bc^				
**Anderson-Fabry Disease (T**_**2**_**) 1.5 T Philips**								
Messalli 2012 [157]	16	81 ± 3			Septum 4CH		1,0,1	Genetically confirmed Anderson-Fabry disease patients.
**1.5 T Siemens**								
Knott 2019 [158]	H+: 24	50.4 ± 3.8^a^	47.5 ± 2.4^c^	< 0.05^ac^	Mean 16 AHA		2,1,2	Anderson-Fabry disease patients classified between with (H+) (maximum wall thickness > 12 mm) and without left ventricular hypertrophy (H-).
	H-: 20/27	47.8 ± 1.7^b^		NS^bc^				
**Hypertrophic Cardiomyopathy (T**_2_^*^) 1.5 T Philips								
Gastl 2019 [159]	LGE: 75	25.2 ± 4.0	31.3 ± 4.3		Septal mid-SAx	FFE	2,2,2	HCM patients classified between with (LGE+) and without LV fibrosis (LGE-).
	LGE-: 20/28	28.7 ± 5.3						
**Hypertrophic Cardiomyopathy (T**_2_^*^) 3 T GE								
Kanzaki 2016 [160]	16/18	22.3 ± 4.1	21.0 ± 6.4		Septal mid-SAx		2,0,2	HCM patients with hypertrophied non-dilated LV (LV wall thickness > 13 mm) without other cardiovascular diseases.
**Hypertrophic Cardiomyopathy (T**_**2**_**) 1.5 T Philips**								
Amano 2015 [161]	21/7	59.8 ± 6.4	48.1 ± 3.2	< 0.01	High T_2_ SAx	GraSE	1,0,2	HCM patients with maximum LV thickness of ≥15 mm and non-dilated LV asymmetrical hypertrophy without other cardiovascular hypertrophy diseases.
**1.5 T Siemens**								
Park 2018 [162]	88	55.5 ± 3.2			Mean 16 AHA	T_2_-prep SSFP	2,0,1	HCM patients with maximal LV hypertrophy ≥13 mm and ratio 1.3 maximal thickness to posterior wall without other cause hypertrophy.
**Dilated Cardiomyopathy (T**_2_^*^) 3 T Philips								
Nagao 2015 [163]	E+: 13	30.0 ± 4.0			Septal mid-SAx	GRE	1,0,2	DCM patients with LVEF < 45% classified between with (E+) and without major adverse cardiac events (E-).
	E-: 33	25.7 ± 4.1						
**3 T GE**								
Kanzaki 2016 [160]	48/18	18.7 ± 3.1	21.0 ± 6.4		Septal mid-SAx		2,0,2	DCM patients diagnosed with World Health Organization criteria.
**Dilated Cardiomyopathy (T**_**2**_**) 1.5 T Philips**								
Ito 2015 [164]	R+: 12	61.4 ± 3.1			Mean 16 AHA	FSE	2,0,1	DCM patients diagnosed with World Health Organization criteria treated by HF guidelines classified as responders (R+) (ΔLVEF > 15% after 6 m) and non-responders (R-).
	R-: 10	68.1 ± 7.9						
Kono 2014 [165]	12	64.5 ± 7.0			3 SAx	FSE	1,0,1	DCM patients diagnosed on clinical, echocardiographic and nuclear medicine findings.
Nishii 2014 [166]	M: 12	61.2 ± 0.4^a^	51.2 ± 1.6^c^	< 0.01^ac^	3 SAx	FSE	3,0,2	Mild DCM patients LVEF > 35% (M), severe DCM ≤ 35% (S).
	S: 14/15	67.4 ± 6.8^b^		< 0.01^bc^				
Spieker 2017 [167]	M: 23	66.2 ± 7.5^a^	60.0 ± 4.2^c^	< 0.01^ac^	Mean 16 AHA	GraSE	1,2,2	Mild DCM patients LVEF > 30% (M), severe DCM ≤ 30% (S).
	S: 34/60	65.5 ± 5.3^b^		< 0.01^bc^				
**1.5 T Siemens**								
Cui 2018 [168]	12/15	50 ± 3	45 ± 1	< 0.01	Mid-wall	T_2_-prep SSFP	3,2,1	DCM patients with LV dilatation, LVEF < 35% and without CAD.
Mordi 2016 [169]	16/21	55.9 ± 4.4	52.9 ± 3.3	< 0.01	Mean septal basal and mid-SAx	T_2_-prep SSFP	2,1,2	DCM patients (LVEF 40–50% by echocardiography).
**Dilated Cardiomyopathy (T**_**2**_**) 3 T Philips**								
Child 2018 [170]	32/26	47 ± 5	45 ± 3		Septal mid-SAx LGE-	GraSE	2,2,2	Non-ischemic DCM patients with LVEF < 50%.
**Myocarditis (T**_**2**_**) 1.5 T Philips**								
Baeßler 2017 [171]	I: 31	62 ± 7^a^	59 ± 4^c^	< 0.05^ac^	Mean 16 AHA	GraSE	3,0,2	Initial cohort (I) of CMR-positive myocarditis patients. Validation cohort (V) of CMR-positive myocarditis (n = 22) + clinically diagnosed (*n* = 31) + no LLC (*n* = 15).
	V: 68/30	64 ± 6^b^		< 0.01^bc^				
Baeßler 2018 [172]	26/10	62.1 ± 4.8	55.8 ± 1.8	< 0.01	Mean HLA & mid-SAx	SE	3,0,2	Acute myocarditis patients with infarct like presentation and positive biventricular EMB.
Baeßler 2019 [173]	AB+: 21	64.3 ± 5.5			Mean HLA & mid-SAx	SE	2,0,1	Myocarditis patients defined as acute (A) (symptoms ≤14d) or chronic (C) and classified based on positive (B+) or negative EMB (B-).
	AB-: 10	60.2 ± 5.8						
	CB+: 26	63.4 ± 5.3						
	CB-: 14	61.1 ± 3.1						
Bohnen 2017 [174]	F0: 48	61.3 ± 4.6^a^	55.0 ± 3.1^b^	< 0.05^ab^	LGE+ in 3 SAx	GraSE	3,0,2	Acute myocarditis patients scanned in acute phase (F0), after 3 months (F1) and after 12 months (F2).
	F1: 39	56.7 ± 4.6						
	F2: 21/27	54.0 ± 4.0						
Bohnen 2015 [175]	16	65.3 ± 7.3			3 SAx	SE	2,0,1	Patients with recent-onset HF, LVEF < 45% without CAD and positive EMB (3d before scan).
Dabir 2019 [176]	50/30	58.0 ± 6.0	51.6 ± 1.9	< 0.01	3 SAx	GraSE	3,0,2	Patients meet diagnostic criteria for clinically acute myocarditis 3d after symptom onset.
Gatti 2019 [177]	8/30	55.7 ± 4.2	46.8 ± 1.6	< 0.01	3 SAx	GraSE	2,0,2	Patients with clinically acute myocarditis and LVEF ≥55%.
Luetkens 2017 [178]	48/35	62.2 ± 8.8	52.3 ± 2.5	< 0.01	3 SAx	GraSE	3,0,2	Patients with acute myocarditis 3d after symptom onset.
Luetkens 2019 [38]	40/26	61.8 ± 8.2	52.8 ± 2.4	< 0.01	3 SAx	GraSE	2,0,2	Patients with clinically defined acute myocarditis 4d after hospital admission.
Lurz 2016 [179]	A: 43	62.2 ± 4.5			1 SAx		1,0,1	Confirmed myocarditis patients classified as acute (A) (acute symptoms ≤14d) or chronic (C) (symptoms >14d).
	C: 48	62.8 ± 4.5						
Radunski 2014 [180]	104/21	61.3 ± 5.3	56.3 ± 4.8	< 0.01	3 SAx		2,0,2	Myocarditis patients 2w (median) after symptom onset.
Radunski 2017 [181]	20/20	97.3 ± 23.1	56.7 ± 4.8	< 0.01	LGE in 3 SAx	SE	2,0,2	Myocarditis patients with positive LLC 3d (median) after symptom onset.
Spieker 2017 [182]	46/60	68.1 ± 5.8	60.0 ± 4.2	< 0.01	Mean 16 AHA	GraSE	2,2,2	Suspected acute myocarditis patients on ESC guidelines 5d after onset.
**1.5 T Siemens**								
Huber 2018 [183]	20/20	53 ± 4^a^	48 ± 2^c^	< 0.05^ac^	Mean basal and mid-SAx	T_2_-prep SSFP	3,0,2	Acute viral myocarditis patients based on clinical guidelines 5d after symptom onset.
Mayr 2017 [184]	39/10	65.3 ± 45.4	53.7 ± 31.0	< 0.01	LGE+ in 3 SAx	TSE	1,0,2	Cardiac disease symptoms, evidence of myocardial injury by elevated serum markers, exclusion of CAD 4d (median) after symptom onset.
Thavendiranathan 2013 [25]	20/30	65.2 ± 3.2	54.5 ± 2.2		LGE+ AHA	T_2_-prep SSFP	3,0,2	Acute myocarditis patients 1d (median) after hospital admission.
Von Knobelsdorff Brenkenhoff 2017 [185]	F0:18	55.2 ± 3.1^a^	50.4 ± 2.3^d^	< 0.01^ad^	Mean basal and mid-SAx	T_2_-prep SSFP	1,2,2	Acute myocarditis patients <7d (F0), 40d (F1) and 189d (F2) after symptom onset.
	F1: 18	52.4 ± 1.0^b^		< 0.01^bd^				
	F2: 18/18	51.3 ± 3.0^c^		0.32^cd^				
**Myocarditis (T**_**2**_**) 3 T Siemens**								
Gang 2019 [186]	35/35	65.5 ± 8.5	55.2 ± 3.6	< 0.05		T_2_-prep SSFP	2,0,2	Clinically suspected myocarditis patients 2.6 ± 1.9d after hospital admission.
Stirrat 2018 [187]	9/10	57.1 ± 5.3	46.7 ± 1.6	< 0.01	LGE+ SAx & LAx	T_2_-prep tFISP	2,0,2	Confirmed acute myocarditis patients 1w after diagnosis.
**Hypertension (T**_2_^*^) 1.5 T Philips								
Chen 2018 [188]	H+: 20	23.8 ± 3.1^a^	30.8 ± 2.7^c^	< 0.05^ac^		TFE	2,0,2	Hypertension patients with (H+) and without (H-) LV hypertrophy.
	H-: 21/23	28.7 ± 4.2^b^		< 0.05^bc^				

*4CH* 4 chamber, *AHA* American Heart Association, *AL* amyloid light-chain, *ATTR* amyloid transthyretin, *ß-TM* beta thalassemia major, *CAD* coronary artery disease, *CMR* cardiovascular magnetic resonance, *D* days, *DCM* dilated cardiomyopathy, *EMB* endomyocardial biopsy, *ESC* European Society of Cardiology, *FFE* fast field echo, *FSE* fast spin echo, *GraSE* gradient spin echo, *GRE* gradient echo, *H* hours, *HCM* hypertrophic cardiomyopathy, *HF* heart failure, *HLA* horizontal long axis, *LAx* long axis, *LGE* late gadolinium enhancement, *LLC* Lake Louis criteria, *LV* left ventricle, *LVEF* left ventricular ejection fraction, *M* months, *MI* myocardial infarction, *NS* non-significant, *NSTEMI* non-ST-elevation myocardial infarction, *PCI* percutaneous coronary intervention, *Qual*. outcome Newcastle-Ottawa quality assessment scale, *ROI* region-of-interest, *SAx* short axis, *SD* standard deviation, *SE* spin echo, *Seq*. MR sequence, *SI* spiral imaging, *SLE* systemic lupus erythematosus, *SSFP* steady-state free precession, *STEMI* ST-elevation myocardial infarction, *T*_*2*_*-prep*. T_2_-prepared, *TFE* turbo field echo, *tFISP* true fast imaging with steady state precession, *TI* thalassemia intermedia, *TM* thalassemia major, *TSE* turbo spin echo, *W* weeks

### Study quality

None of the included studies received the maximum NOS quality score (Table 1). All studies without healthy controls automatically received limited scores in the matching and selection section. Only 57 of the 154 included studies reported control values of healthy subjects. The case definition of patients and the ascertainment of mapping values were adequate in all studies.

### Myocardial infarction

**Fig. 2:**
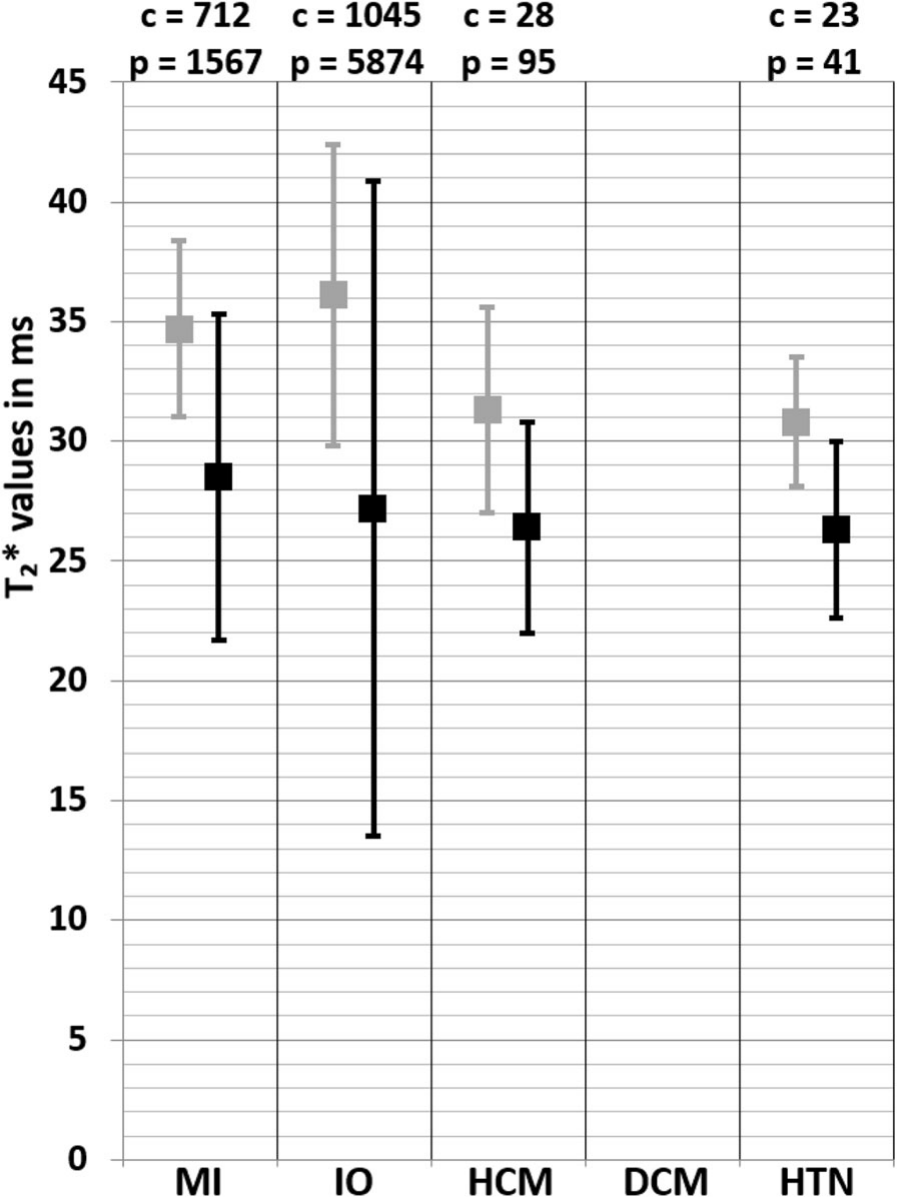
Weighted mean T_2_^*^ values and weighted standard deviations (SD) of all included papers reporting T_2_^*^ values of both patients (black squares) and controls (grey squares) measured at 1.5 T. The number of included patient (p) and control (c) measurements for each population is reported above the graph. MI myocardial infarction, IO iron overload, HCM hypertrophic cardiomyopathy, DCM dilated cardiomyopathy, HTN hypertension

**Fig. 3:**
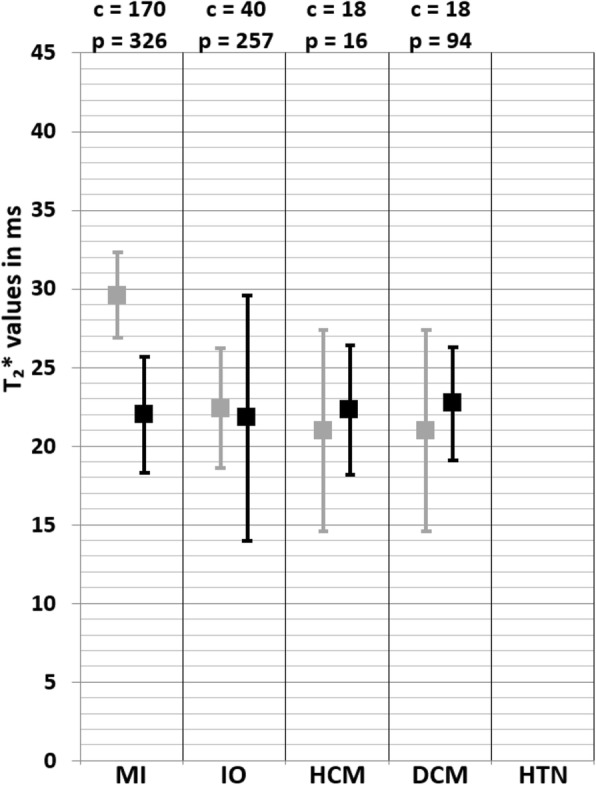
Weighted mean T_2_^*^ values and weighted standard deviations (SD) of all included papers reporting T_2_^*^ values of both patients (black squares) and controls (grey squares) measured at 3 T. The number of included patient (p) and control (c) measurements for each population is reported above the graph. MI myocardial infarction, IO iron overload, HCM hypertrophic cardiomyopathy, DCM dilated cardiomyopathy, HTN hypertension

**Fig. 4:**
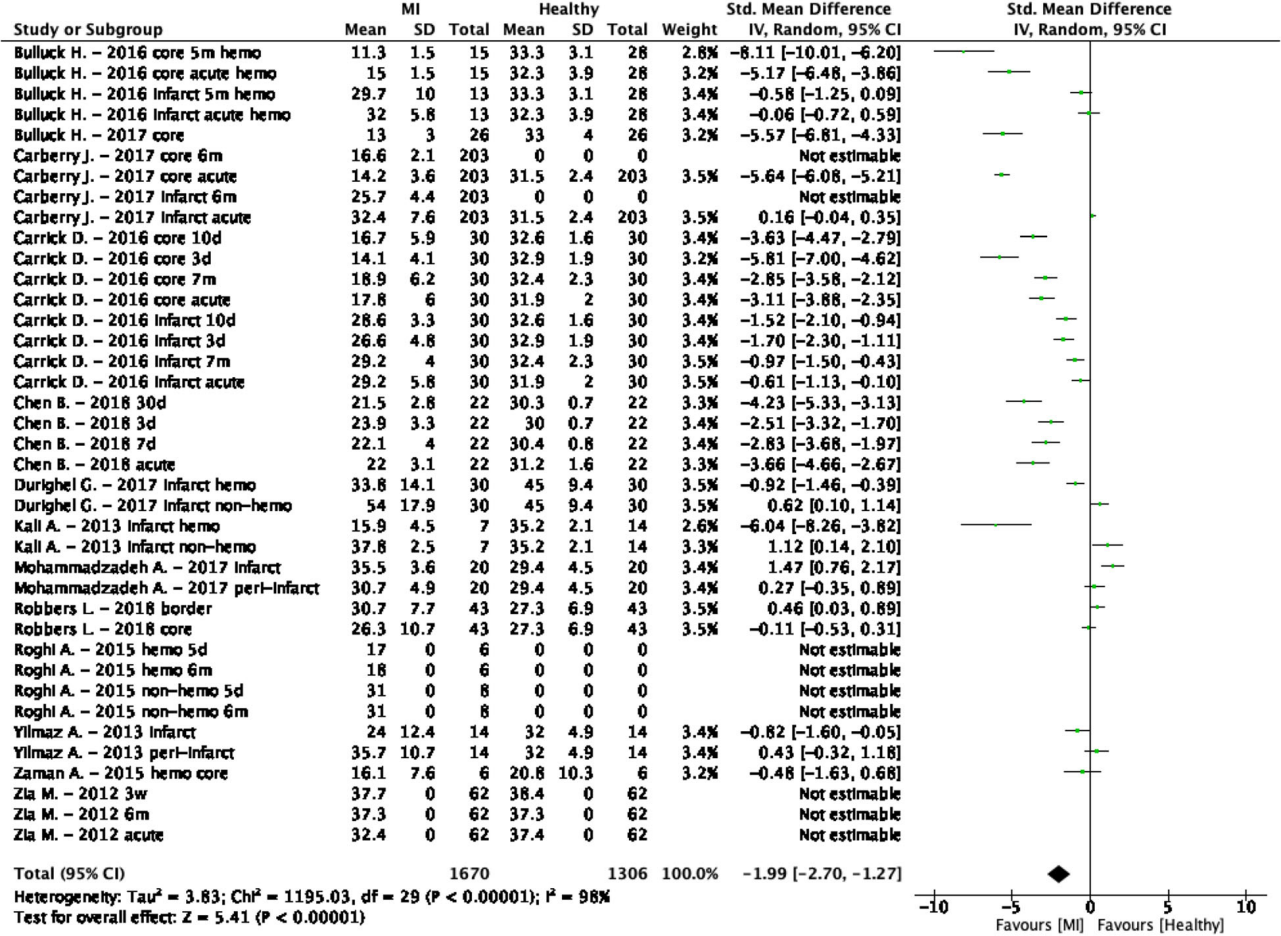
Standardized mean differences between T_2_^*^ of myocardial infarction (MI) patients and healthy controls with associated random effects weight factors. CI confidence interval, IV inverse variance

The weighted mean T_2_^*^ values at 1.5 T in myocardial infarction (MI) patients was 28.5 ± 6.8 ms and 34.7 ± 3.7 ms in controls [39–49] (Table 1, Fig. 2). At 3 T, these were 22.0 ± 3.7 ms in MI patients and 29.6 ± 2.7 ms in controls [50, 51] (Table 1, Fig. 3). The meta-analysis confirmed significantly lower T_2_^*^ values in MI patients (SMD = − 1.99, 95% Cl [− 2.70, − 1.27], *P* <  0.01, I^2^ = 98%, Fig. 4). Most studies performed CMR in ST-elevation myocardial infarction (STEMI) patients post percutaneous coronary intervention (PCI) in the acute phase [39–44, 46–51]. Some studies performed follow-up in these patient groups [42–44, 47, 49, 50] and Mohammadzadeh et al. [45] was the only study including non-STEMI (NSTEMI) patients. Most studies reported T_2_^*^ values of multiple regions-of-interest (ROI) in the myocardium (Table 1). Although none of the tested covariates was significant, the difference in T_2_^*^ values seemed larger in the infarct cores compared to the infarct zone as a whole. Significant funnel asymmetry was found for the random effects model suggesting eight missing studies with negative results (*P* <  0.01), while the mixed effects model did not show funnel asymmetry (*P* = 0.60).

The heterogeneity was not corrected with the existing covariates and therefore a second analysis was performed where the reported T_2_^*^ values were divided in infarct zone or infarct core groups. The infarct zone, which is determined by LGE, is the affected myocardium characterized by edema excluding the hypo-intense core, which is the center in the infarct zone with T_2_^*^ values < 20 ms identifying the presence of hemorrhage [40, 50]. Although during myocardial infarction no haemorrhagic core is present, the patients were referred for CMR after PCI in most studies. The process of reperfusion after PCI frequently leads to simultaneous microvascular obstruction and intramyocardial haemorrhage within the infarct zone [41, 191].

Eight studies [39–41, 43–45, 48, 50] explicitly reported infarct zone values. The weighted mean T_2_^*^ value at 1.5 T of the infarct zones was 32.3 ± 5.4 ms and at 3 T this was 22.4 ± 2.8 ms (Fig. 1, Supplementary Data 2). These T_2_^*^ values also resulted in significantly lower values compared to controls (SMD = − 1.21, 95% Cl [− 1.83, − 0.59], *P* <  0.01, I^2^ = 95%), and with a significant heterogeneity. Furthermore, infarct core values were explicitly reported in five studies [40, 41, 43, 46, 51]. The weighted mean T_2_^*^ value at 1.5 T of infarct cores was 16.1 ± 4.2 ms and at 3 T this was 16.1 ± 7.6 ms (Fig. 1, Supplementary Data 2). These infarct core values showed a larger SMD (SMD = − 4.00, 95% Cl [− 5.67, − 2.32], *P* <  0.01, I^2^ = 98%), while the heterogeneity remained significant. Multiple studies reported the remote myocardium as control which had a weighted mean T_2_^*^ value at 1.5 T of 34.0 ± 4.9 ms and 30.5 ± 1.0 ms at 3 T (Fig. 1, Supplementary Data 2).

**Fig. 5:**
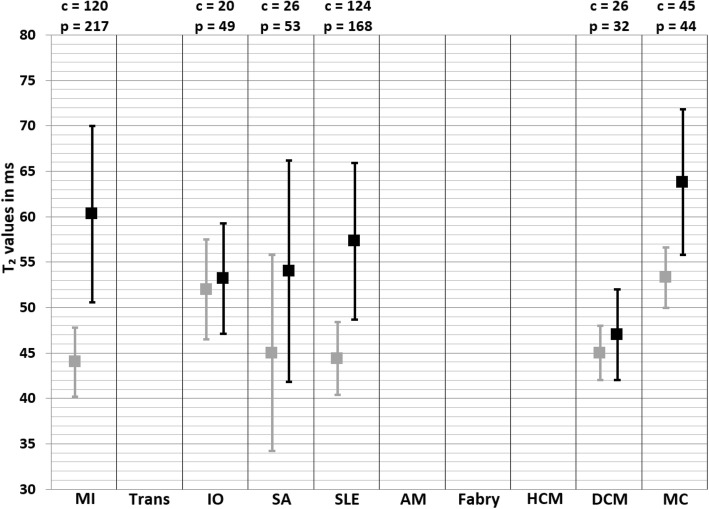
Weighted mean T_2_ values and weighted standard deviations (SD) of all included papers reporting T_2_ values of both patients (black squares) and controls (grey squares) measured at 1.5 T. The number of included patient (p) and control (c) measurements for each population is reported above the graph. MI myocardial infarction, Trans heart transplant, IO iron overload, SA sarcoidosis, SLE systemic lupus erythematosus, AM amyloidosis, HCM hypertrophic cardiomyopathy, DCM dilated cardiomyopathy, MC myocarditis

**Fig. 6:**
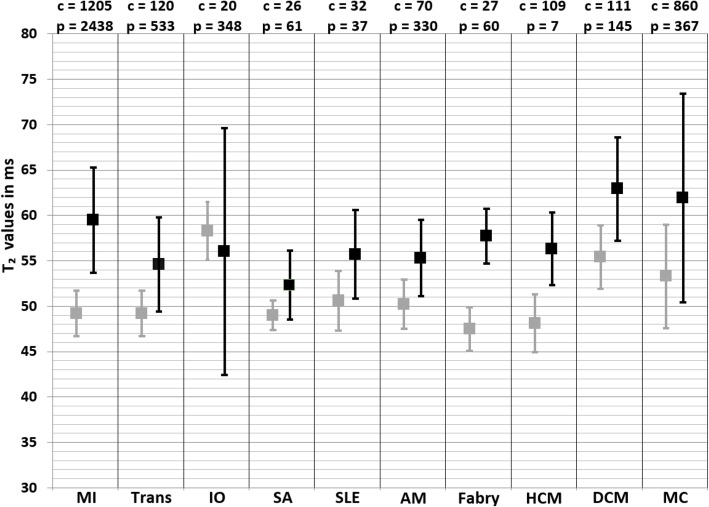
Weighted mean T_2_ values and weighted standard deviations (SD) of all included papers reporting T_2_ values of both patients (black squares) and controls (grey squares) measured at 3 T. The number of included patient (p) and control (c) measurements for each population is reported above the graph. MI myocardial infarction, Trans heart transplant, IO iron overload, SA sarcoidosis, SLE systemic lupus erythematosus, AM amyloidosis, HCM hypertrophic cardiomyopathy, DCM dilated cardiomyopathy, MC myocarditis

**Fig. 7:**
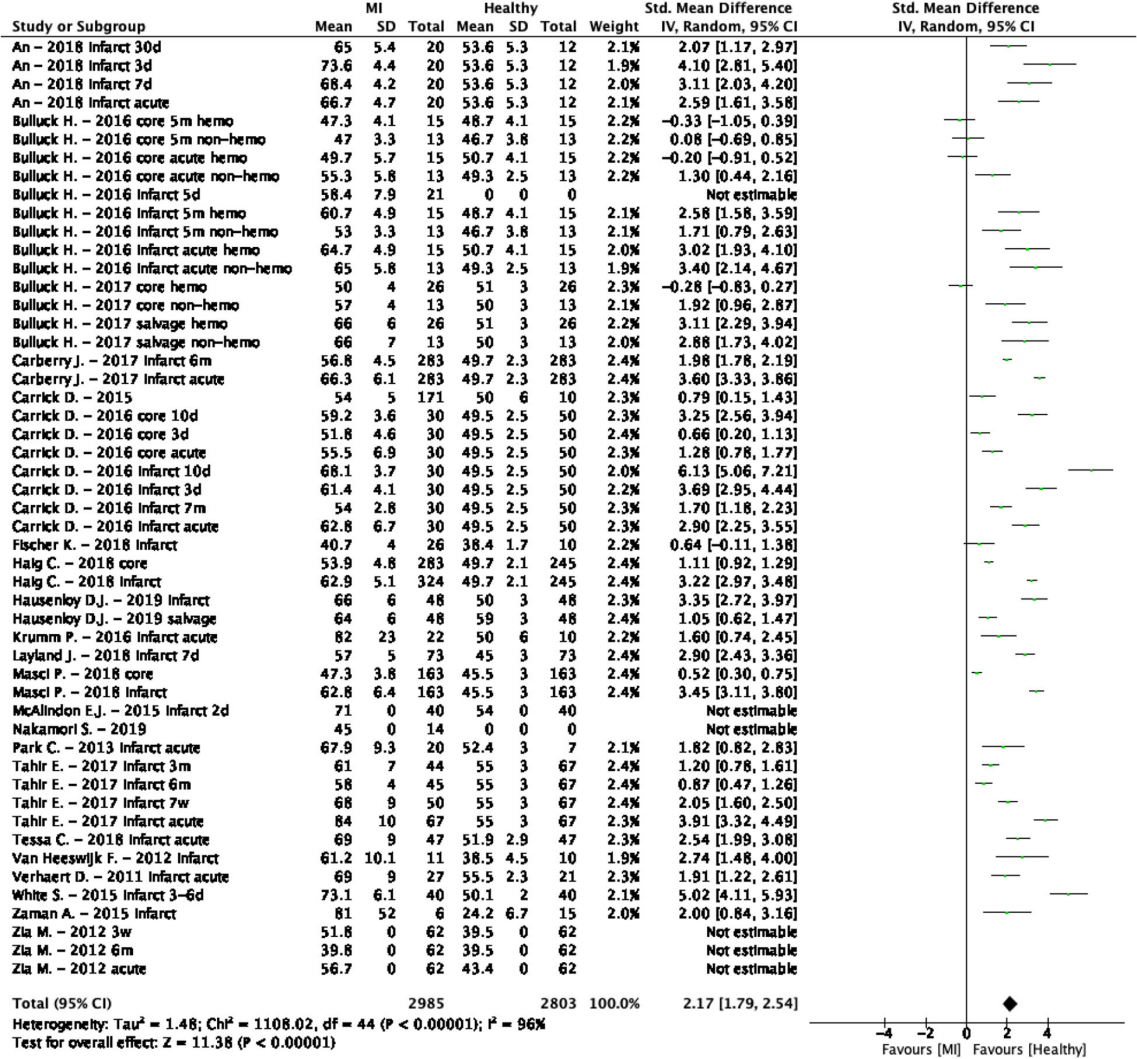
Standardized mean differences between T_2_ of myocardial infarction (MI) patients and healthy controls with associated random effects weight factors. CI confidence interval, IV inverse variance

The weighted mean T_2_ values at 1.5 T in MI patients was 58.5 ± 5.8 ms and 49.3 ± 2.6 ms in controls [26, 40, 41, 43, 49, 52–63] (Table 1, Fig. 5). At 3 T, these were 60.3 ± 9.7 ms in MI patients and 44.0 ± 3.8 ms in controls [51, 64–68] (Table 1, Fig. 6). Most studies restricted their inclusion to STEMI patients [40, 41, 43, 49, 51, 54–60, 63–65, 68], however some studies included specifically NSTEMI patients [52, 62, 67] and others included both STEMI and NSTEMI patients [26, 53, 61, 66]. Besides two studies [52, 62], patients in all studies underwent CMR post-PCI in the acute phase and a few studies also included follow-up data [40, 42, 43, 49, 53, 64]. T_2_ values of different ROIs in the myocardium were reported (Table 1), nevertheless all studies showed higher T_2_ values in all ROIs of MI patients except for studies reporting values of the hemorrhagic core [40, 41]. The meta-analysis confirmed significantly higher T_2_ values in MI patients (SMD = 2.17, 95% CI [1.79, 2.54], *P* <  0.01, I^2^ = 96%, Fig. 7). The age and percentage of men in the control group, the time between intervention and the CMR, the field strength, the type of control (remote myocardium versus healthy controls), the type of CMR acquisition sequence, the ROI location and the left ventricular ejection fraction (LVEF) in patients were significant covariates. There were no other significant residual factors remaining that accounted for the high remaining heterogeneity (I^2^ = 78%), though there are probably other covariates which were not tested due to insufficient data. Publication bias was detected with five possibly missing studies, however no significant asymmetry was found for either the random effects model (*P* = 0.10) or the mixed effects model (*P* = 0.55).

The ROI location was one of the covariates and therefore an additional analysis was performed where the reported T_2_ values were divided in infarct zone and infarct core groups. Infarct zone T_2_ values were reported in 18 studies [26, 40, 43, 51, 53, 54, 56–58, 60–68]. The weighted mean T_2_ value at 1.5 T of infarct zones was 63.7 ± 6.4 ms and at 3 T this was 63.5 ± 10.5 ms (Fig. 2, Supplementary Data 2). The difference between patients and controls was larger when considering only the infarct zone values (SMD = 2.63, 95% Cl [2.25, 3.01], *P* <  0.01, I^2^ = 93%). The meta-analysis showed older patients, a short period between intervention and CMR, lower LVEF in patients and performing CMR on 1.5 T to increase the difference with controls. The used CMR acquisition sequence was also found as significant covariate, nevertheless none of the specified sequences provided clearly larger differences. There were no other significant residual factors remaining that accounted for the heterogeneity (I^2^ = 80%). Again, publication bias was found with two missing studies, however no significant asymmetry was found for either the random effects model (*P* = 0.76) or the mixed effects model (*P* = 0.58). Core T_2_ values were reported in five studies [40, 41, 43, 56, 60]. The weighted mean T_2_ value at 1.5 T of infarct cores was 51.9 ± 4.6 ms and at 3 T no values were reported (Fig. 2, Supplementary Data 2). Including only the T_2_ values of the infarct cores resulted in a smaller difference between patients and controls (SMD = 0.83, 95% Cl [0.37, 2.44], *P* <  0.01, I^2^ = 91%). The weighted mean T_2_ value at 1.5 T of remote myocardium was 49.2 ± 2.5 ms and at 3 T this was 45.0 ± 3.0 ms (Fig. 2, Supplementary Data 2).

### Heart transplant

**Fig. 8:**
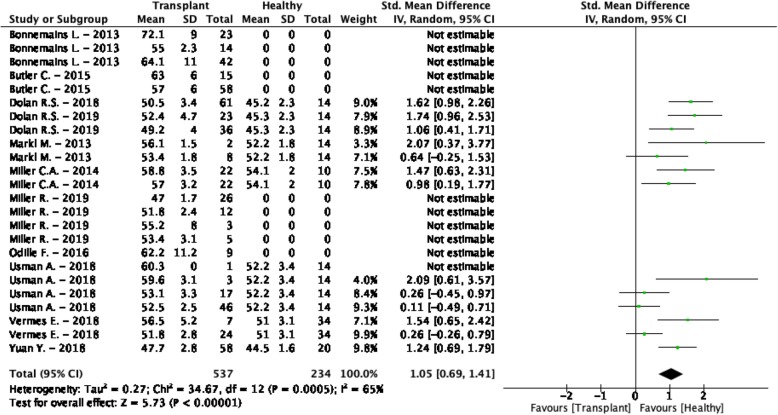
Standardized mean differences between T_2_ of heart transplant patients and healthy controls with associated random effects weight factors. CI confidence interval, IV inverse variance

The weighted mean T_2_ values at 1.5 T in heart transplant patients was 54.6 ± 5.2 ms and 49.2 ± 2.5 ms in controls [27, 69–78] (Table 1, Fig. 5). All studies showed higher T_2_ values in patients compared to controls, only for all subgroups including patients with positive rejection biopsy these values were significantly higher. This meta-analysis confirmed significantly higher T_2_ values in the myocardium of heart transplant patients (SMD = 1.05, 95% CI [0.69, 1.41], *P* <  0.01, I^2^ = 65%, Fig. 8). An exploratory meta-regression analysis indicated that the rejection status, the LVEF and patient age caused the heterogeneity without remaining significant residual factors (I^2^ = 1%). Transplant rejection, lower LVEF and older patients resulted in larger differences between patients and controls.

The cardiac transplant rejection was a significant covariate and therefore the population was divided between positive and negative rejection biopsies. The weighted mean T_2_ values in patients with a positive biopsy [27, 69, 71, 73–75] was 56.4 ± 3.3 ms and 52.5 ± 3.9 ms in patients with a negative biopsy [27, 69, 71–76] (Fig. 2, Supplementary Data 2). None of the studies to heart transplantation described T_2_ values acquired at 3 T or reported T_2_^*^ values.

### Iron overload

**Fig. 9:**
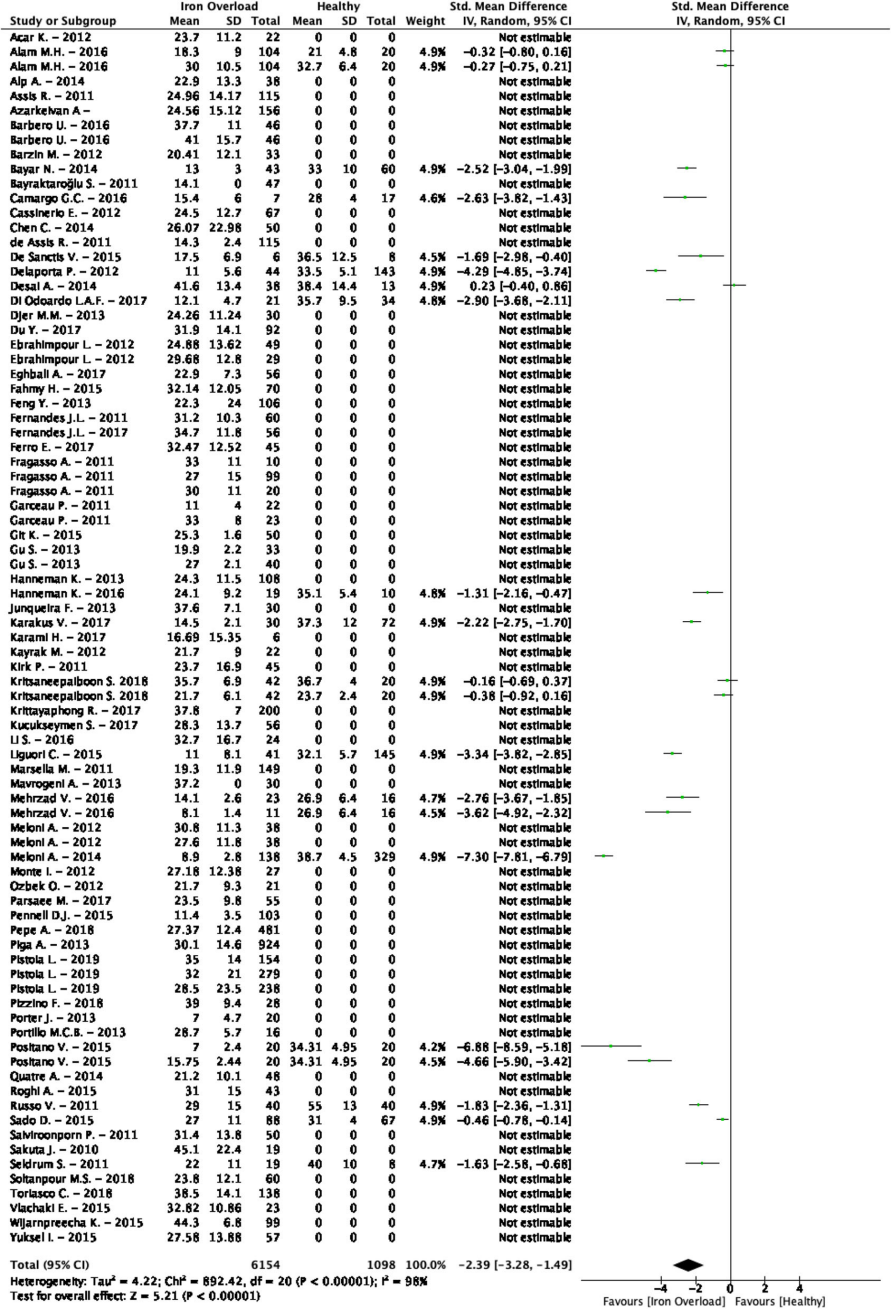
Standardized mean differences between T_2_^*^ of iron overload patients and healthy controls with associated random effects weight factors. CI confidence interval, IV inverse variance

The weighted mean T_2_^*^ values at 1.5 T in iron overload patients was 27.2 ± 13.7 ms and 36.1 ± 6.3 ms in controls [79–147] (Table 1, Fig. 2). At 3 T, these were 21.8 ± 7.8 ms in iron overload patients and 22.4 ± 3.8 ms in controls [81, 88, 127, 148] (Table 1, Fig. 3). The meta-analysis confirmed significantly lower T_2_^*^ values in iron overload patients (SMD = − 2.39, 95% CI [− 3.28, − 1.49], *P* <  0.01, I^2^ = 98%, Fig. 9). The patient populations contained iron overload patients with both cardiac involvement (T_2_^*^ < 20 ms) and without cardiac involvement (T_2_^*^ ≥ 20 ms). Each study that included both iron overload patients and controls showed significantly lower T_2_^*^ values in patients [85, 93, 95, 96, 104, 107, 113, 114, 118, 124, 128, 132, 133, 136, 139], except for two studies that showed non-significant lower T_2_^*^ values [81, 88] and one study that showed non-significantly higher T_2_^*^ values in patients compared to controls [79]. The type of control was found as a covariate which meant using non-cardiac involved iron overload subjects as controls caused larger differences with patients than using healthy controls. The type of patients was also found as covariate; using a population with proven cardiac involvement caused larger differences with controls than using a mix of non-cardiac and cardiac involved iron overload patients. Furthermore, the number of echoes used in the T_2_^*^ sequence was determined as a covariate. These covariates, however, only partly accounted for the heterogeneity in the mixed effects model (I^2^ = 80%), while other tested regressors (age of patient and control population, percentage of men in patient and control population, CMR vendor, field strength and the serum ferritin concentration in patients) had no significant influence. Based on the high remaining heterogeneity there should be other covariates which were not tested due to insufficient data. Significant funnel asymmetry (*P* <  0.01) was only found for the random effects model suggesting five missing studies with populations showing higher T_2_^*^ values compared to healthy subjects.

The type of iron overload patient was one of the covariates and therefore an additional analysis was performed on T_2_^*^ values from cardiac involved iron overload patients (T_2_^*^ < 20 ms) [93, 95, 96, 104, 113, 114, 123, 124, 128, 132, 136, 139, 143, 145]. The weighted mean T_2_^*^ value at 1.5 T in cardiac involved iron overload patients was 11.8 ± 3.7 ms and at 3 T no T_2_^*^ values were reported (Fig. 1, Supplementary Data 2). This analysis also showed significantly lower T_2_^*^ values for cardiac involved iron overload patients compared to controls (SMD = − 3.59, 95% CI [− 4.69, − 2.48], *P* <  0.01, I^2^ = 97%) and this difference was also larger than controls compared to the overall iron overload population.

**Fig. 10:**
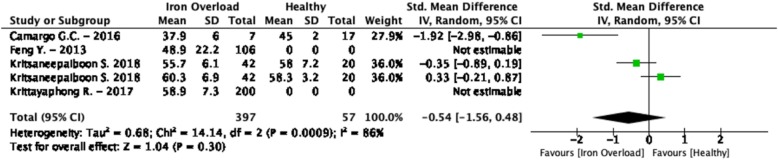
Standardized mean differences between T_2_ of iron overload patients and healthy controls with associated random effects weight factors. CI confidence interval, IV inverse variance

The weighted mean T_2_ values at 1.5 T in iron overload patients was 56.0 ± 13.6 ms and 58.3 ± 3.2 ms in controls [81, 82, 101] (Table 1, Fig. 5). At 3 T, these were 53.2 ± 6.2 ms in iron overload patients and 52.0 ± 5.5 ms in controls [81, 93] (Table 1, Fig. 6). Kritsaineeboon et al. [81] reported no significant changes in T_2_ values for iron overload patients at both 1.5 T and 3 T, while Camargo et al. [93] reported lower T_2_ values in iron overload patients at 1.5 T. The random effects models of all studies combined resulted in no significantly lower T_2_ values for iron overload patients compared to controls (SMD = − 0.54, 95% Cl [− 1.56, 0.48], *P* = 0.30, I^2^ = 86%, Fig. 10).

### Sarcoidosis

**Fig. 11:**
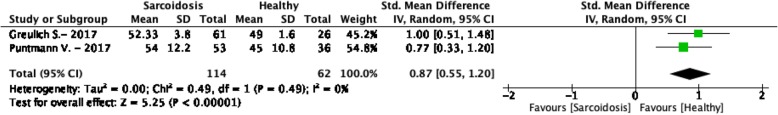
Standardized mean differences between T_2_ of sarcoidosis patients and healthy controls with associated random effects weight factors. CI confidence interval, IV inverse variance

The weighted mean T_2_ values at 1.5 T in sarcoidosis patients was 52.3 ± 3.8 ms and 49.0 ± 1.6 ms in controls [149] (Table 1, Fig. 5). At 3 T, these were 54.0 ± 12.2 ms in sarcoidosis patients and 45.0 ± 10.8 ms in controls [150] (Table 1, Fig. 6). This suggested higher T_2_ values in sarcoidosis patients (SMD = 0.87, 95% CI [0.55, 1.20], *P* <  0.01, I^2^ = 0%, Fig. 11). Insufficient studies were available for further analysis regarding covariates and publication bias, and there was no data that described T_2_^*^ values.

### Systemic lupus erythematosus

**Fig. 12:**
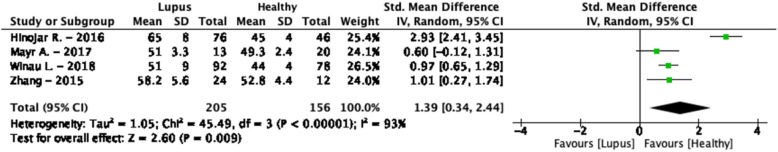
Standardized mean differences between T_2_ of systemic lupus erythematosus patients and healthy controls with associated random effects weight factors. CI confidence interval, IV inverse variance

The weighted mean T_2_ values at 1.5 T in systemic lupus erythematosus (SLE) patients was 55.7 ± 4.9 ms and 50.6 ± 3.3 ms in controls [151, 152] (Table 1, Fig. 5). At 3 T, these were 57.3 ± 8.6 ms in SLE patients and 44.4 ± 4.0 ms in controls [153, 154] (Table 1, Fig. 6). This suggested higher T_2_ values in SLE patients (SMD = 1.39, 95% CI [0.34, 2.44], *P* <  0.01, I^2^ = 93%, Fig. 12). Insufficient studies were available for further analysis regarding covariates and publication bias, and there were no data that described T_2_^*^ values.

### Amyloidosis

**Fig. 13:**
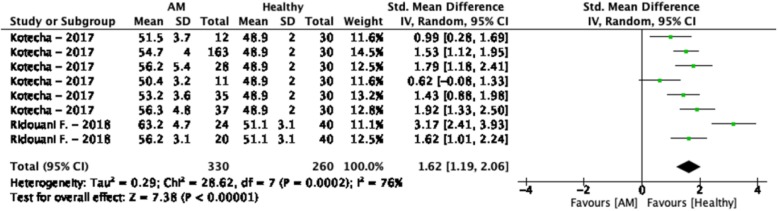
Standardized mean differences between T_2_ of amyloidosis (AM) patients and healthy controls with associated random effects weight factors. CI confidence interval, IV inverse variance

The weighted mean T_2_ values at 1.5 T in amyloidosis patients was 55.3 ± 4.2 ms and 50.2 ± 2.7 ms in controls [155, 156] (Table 1, Fig. 5). All included studies reported higher T_2_ values in amyloidosis patients (SMD = 1.62, 95% CI [1.19, 2.06], *P* <  0.01, I^2^ = 76%, Fig. 13). Although insufficient studies were available for further analysis regarding covariates and publication bias, both included studies reported higher T_2_ values in amyloid light-chain amyloidosis than in transthyretin amyloidosis. Furthermore, there were no studies performed with T_2_ values on 3 T and there was no data that described T_2_^*^ values.

### Anderson-Fabry disease

**Fig. 14:**
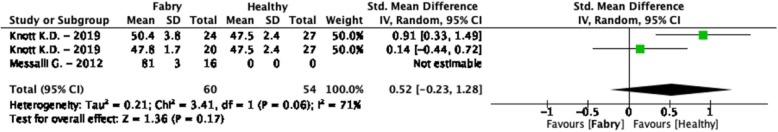
Standardized mean differences between T_2_ of Fabry disease patients and healthy controls with associated random effects weight factors. CI confidence interval, IV inverse variance

The weighted mean T_2_ value at 1.5 T in Anderson-Fabry disease patients was 57.7 ± 3.0 ms [157, 158] (Table 1, Fig. 5). One study reported T_2_ values in controls of 47.5 ± 2.4 ms [158], suggesting a trend to higher T_2_ values in Anderson-Fabry disease patients (SMD = 0.52, 95% Cl [− 0.23, 1.28], *P* = 0.17, I^2^ = 71%, Fig. 14). The higher T_2_ values in Anderson-Fabry disease patients were caused by the reported T_2_ values in Anderson-Fabry disease patients with left ventricular hypertrophy (LVH) (50.4 ± 3.8 ms), while patients without LVH showed similar T_2_ values (47.8 ± 1.7 ms) to controls. Insufficient studies were available for further analysis regarding covariates and publication bias. Furthermore, there were no studies performed with T_2_ values on 3 T and there were no data that described T_2_^*^ values.

### Hypertrophic cardiomyopathy

**Fig. 15:**
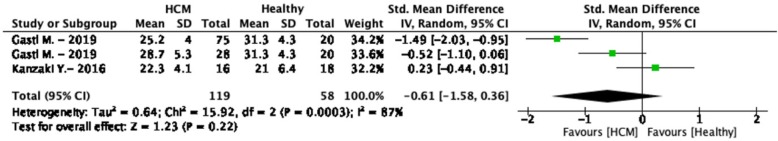
Standardized mean differences between T_2_^*^ of hypertrophic cardiomyopathy (HCM) patients and healthy controls with associated random effects weight factors. CI confidence interval, IV inverse variance

The weighted mean T_2_^*^ values at 1.5 T in HCM patients from one study was 26.4 ± 4.4 ms and 31.3 ± 4.3 ms in controls [159] (Table 1, Fig. 2). At 3 T, these were 22.3 ± 4.1 ms in HCM patients and 21.0 ± 6.4 ms in controls [160] (Table 1, Fig. 3). The study performed at 1.5 T reported values in subgroups based on the presence of fibrosis (with or without LGE) and in both subgroups the T_2_^*^ value was lower compared to controls, which was only significant in the group with LGE presence [159]. In the study performed at 3 T there, however, was no significant difference in T_2_^*^ values between HCM patients with or without LGE presence. As result, the analysis showed a no significant difference between HCM patients and controls (SMD = − 0.61, 95% CI [− 1.58, 0.36], *P* = 0.22, I^2^ = 87%, Fig. 15). Insufficient studies were available for further analysis regarding covariates and publication bias.

**Fig. 16:**
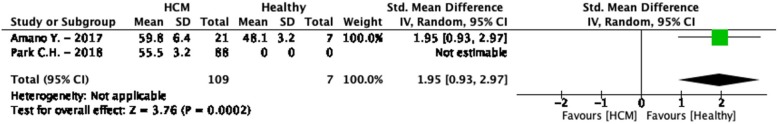
Standardized mean differences between T_2_ of hypertrophic cardiomyopathy (HCM) patients and healthy controls with associated random effects weight factors. CI confidence interval, IV inverse variance

The weighted mean T_2_ value at 1.5 T in HCM patients was 56.3 ± 4.0 ms [161, 162] (Table 1, Fig. 5). One study reported T_2_ values in controls of 48.1 ± 3.2 ms suggesting significantly higher T_2_ values in HCM patients [161] (SMD = 1.95, 95% Cl [0.93, 2.97], I^2^ = N/A, *P* <  0.01, Fig. 16). In that same study [161] the T_2_ values were measured in the patient myocardium with visually high T_2_, which was present in 38% of the patients. For the patients without LGE in that study the myocardial T_2_ value of 48.8 ± 2.4 ms was not significantly different from controls. Furthermore, there were no studies performed with T_2_ values acquired at 3 T and insufficient studies were available for further analysis regarding covariates and publication bias.

### Dilated cardiomyopathy

**Fig. 17:**
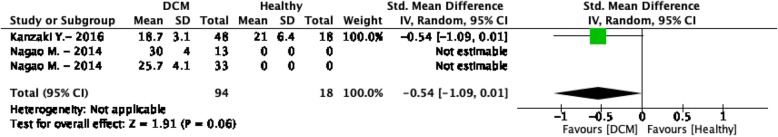
Standardized mean differences between T_2_^*^ of dilated cardiomyopathy (DCM) patients and healthy controls with associated random effects weight factors. CI confidence interval, IV inverse variance

The weighted mean T_2_^*^ value at 3 T in DCM patients was 22.7 ± 3.6 ms [160, 163] and only one of those studies reported T_2_^*^ values in controls of 21.0 ± 6.4 ms [160] (Table 1, Fig. 3). The random effects model was therefore only based on that study, and since that study reported T_2_^*^ values of 18.7 ± 3.1 ms in DCM patients there was no significant change in T_2_^*^ values (SMD = − 0.54, 95% Cl [− 1.09, 0.01], I^2^ = N/A, *P* = 0.06, Fig. 17). In both studies, patients had chronic established DCM and without myocarditis or other cardiomyopathies [160, 163]. Furthermore, there were no studies performed with T_2_^*^ values acquired at 1.5 T and there were also insufficient studies available for further analysis regarding covariates and publication bias.

**Fig. 18:**
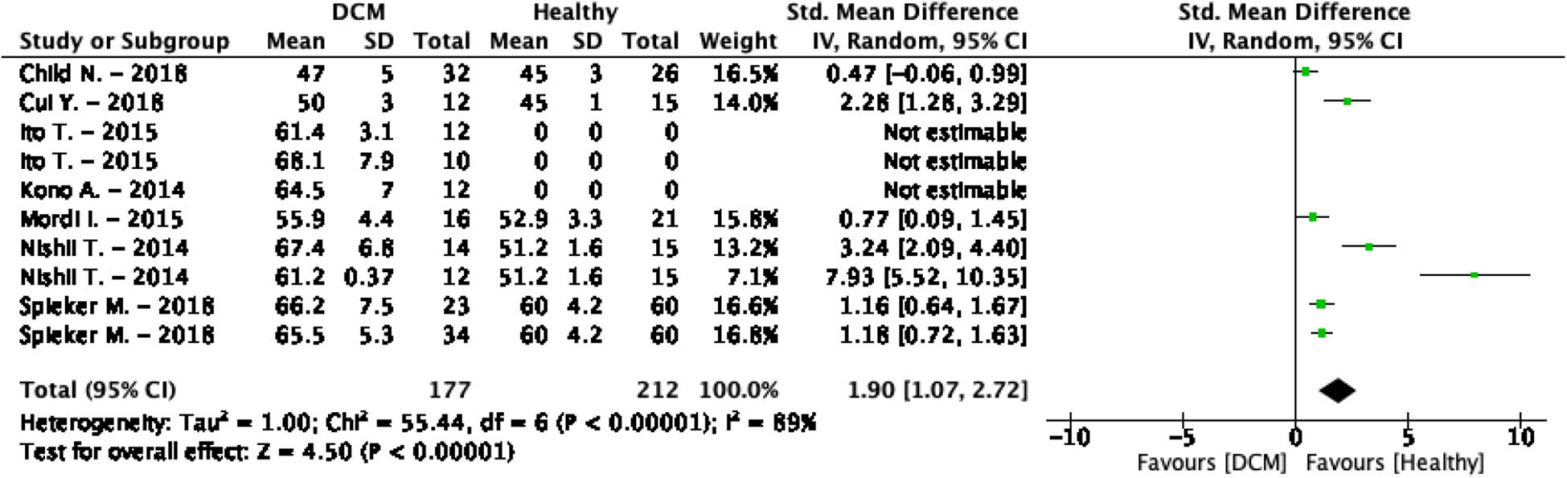
Standardized mean differences between T_2_ of dilated cardiomyopathy (DCM) patients and healthy controls with associated random effects weight factors. CI confidence interval, IV inverse variance

The weighted mean T_2_ values at 1.5 T in DCM patients was 62.9 ± 5.7 ms and 55.4 ± 3.5 ms in controls [164–169] (Table 1, Fig. 5). At 3 T, these were 47.0 ± 5.0 ms in DCM patients and 45.0 ± 3.0 ms in controls [170] (Table 1, Fig. 6). All studies reported significantly higher T_2_ values in DCM patients compared to controls, except for the single study performed at 3 T [170]. The similar T_2_ values of patients and controls in this study might be related to their ROI placement, since they explicitly excluded positive LGE segments from the ROI, while all other studies used the entire myocardium without excluding positive LGE segments [164–169]. Nevertheless, the T_2_ values of positive and negative LGE segments were similar in all studies that reported T_2_ values of both segments [166–168]. The overall meta-analysis confirmed the significantly higher T_2_ values in DCM patients (SMD = 1.90, 95% CI [1.07, 2.72], *P* <  0.01, I^2^ = 89%, Fig. 18) and an exploratory meta-regression analysis indicated the MR vendor and the age difference between DCM patients and controls as possible covariates. The use of a Philips Healthcare CMR scanner and a bigger age difference between control and patient groups resulted in a larger SMD between DCM patients and controls.

### Myocarditis

**Fig. 19:**
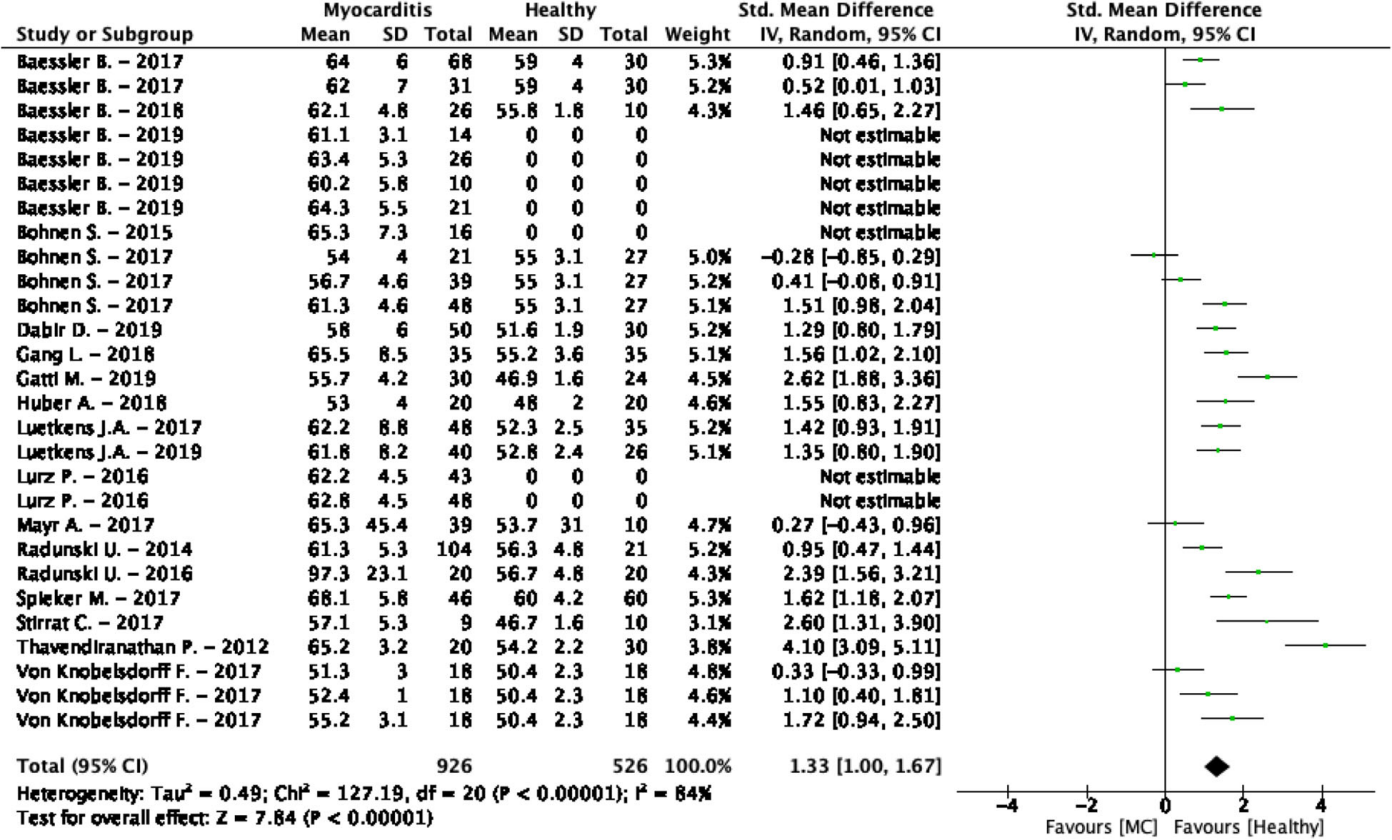
Standardized mean differences between T_2_ of myocarditis (MC) patients and healthy controls with associated random effects weight factors. CI confidence interval, IV inverse variance

The weighted mean T_2_ values at 1.5 T in myocarditis patients was 61.9 ± 11.5 ms and 54.4 ± 5.9 ms in controls [25, 38, 171–185] (Table 1, Fig. 5). At 3 T, these were 63.8 ± 8.0 ms in myocarditis patients and 53.3 ± 3.3 ms in controls [186, 187] (Table 1, Fig. 6). The meta-analysis confirmed significantly higher T_2_ values in myocarditis patients (SMD = 1.33, 95% CI [1.00, 1.67], *P* <  0.01, I^2^ = 84%, Fig. 19). Multiple significant covariates were identified including; the difference in LVEF between patients and controls, the difference in percentage men between patients and controls, the time between symptoms and CMR, the number of echoes used in the CMR acquisition sequence, the CMR vendor and the slice thickness. These covariates together corrected for the total heterogeneity (I^2^ = 0%) and resulted in a larger SMD between myocarditis patients and controls when the same percentages of men was used in both groups, a significantly decreased LVEF was seen in patients, six echoes were acquired for the mapping, a Siemens Healthineers CMR vendor was used, a bigger slice thickness was used, and when the patients were scanned in the acute phase of myocarditis. Significant asymmetry was not found for either the random effects model (*P* = 0.12) or the mixed effects model (*P* = 0.10).

The time between symptom onset and CMR was found as significant covariate and therefore the population was divided between T_2_ values from patients in the acute phase and non-acute phase [192]. Acute myocarditis in patients was diagnosed using the European Society of Cardiology guideline [193] and these patients were referred for CMR shortly after symptom onset in the acute phase (< 14 days). Myocarditis patients in the non-acute phase either had chronic symptom duration (> 14 days) or underwent CMR follow-up. The weighted T_2_ value of myocarditis patients in the acute phase at 1.5 T was 63.5 ± 15.0 ms and at 3 T this was 63.8 ± 8.0 ms [25, 38, 167, 172–179, 181, 183–187] (Fig. 2, Supplementary Data 2). The weighted T_2_ value of myocarditis patients in the non-acute phase at 1.5 T was 58.3 ± 4.3 ms [173, 174, 179, 185] and at 3 T no T_2_ values were reported (Fig. 2, Supplementary Data 2). Furthermore, there were no studies that described T_2_^*^ values for myocarditis.

### Hypertension

**Fig. 20:**
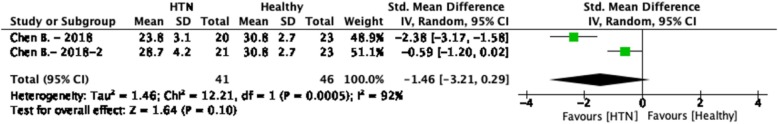
Standardized mean differences between T_2_^*^ of hypertension (HTN) patients and healthy controls with associated random effects weight factors. CI confidence interval, IV inverse variance

One study reported T_2_^*^ values at 1.5 T in hypertension patients of 26.3 ± 3.7 ms and 30.8 ± 2.7 ms in controls [188] (Table 1, Fig. 2). This suggested lower T_2_^*^ values in hypertension patients, nevertheless this was not significant (SMD = − 1.46, 95% CI [− 3.21, 0.29], *P* = 0.10, I^2^ = 92%, Fig. 20). This study classified the included hypertension population in either presence of LVH or no presence of LVH, and showed in both subgroups lower T_2_^*^ values, however in hypertension patients with LVH the T_2_^*^ values were lowest. Furthermore, insufficient studies were available for further analysis regarding covariates and publication bias, and there were no studies that described T_2_^*^ values acquired at 3 T or T_2_ results. Also, no published data was found on T_2_ or T_2_^*^ for the cardiovascular risk populations obesity and diabetes.

## Discussion

Quantitative analysis of factors that modulate myocardial T_2_ and T_2_^*^, such as edema, lipids and paramagnetic iron-containing depositions, can potentially provide additional diagnostic information to distinguish between myocardial diseases and healthy myocardium. This meta-analysis confirmed that T_2_ mapping can help differentiate between healthy subjects and patients affected by MI, DCM, myocarditis or heart transplantation, since T_2_ values were higher in these populations [22]. Although T_2_ mapping has been expected to be sensitive to iron as well [22], no significantly lower T_2_ values were found between iron overload related diseases and healthy myocardium (*P* = 0.30). On sarcoidosis, SLE, amyloidosis, sarcoidosis, Anderson-Fabry disease and HCM insufficient studies were reported for further analysis, nevertheless the available data suggested T_2_ values to be higher within these diseases, with an exception for Anderson-Fabry disease patients without LVH. Furthermore, this meta-analysis confirmed that T_2_^*^ mapping can differentiate between healthy myocardium and myocardium affected in MI and iron overload, since T_2_^*^ values were lower in both of these populations [22]. For HCM, DCM and hypertension patients, the limited available T_2_^*^ mapping studies also gave some indication of lower T_2_^*^ values compared to controls, however this was overall not significant. For all included cardiac diseases in this meta-analysis the T_2_ values were higher, with iron overload patients as an exception showing lower T_2_ values, and T_2_^*^ values were lower. These similarities in T_2_ and T_2_^*^ values between cardiac diseases prevent further differentiation in disease type, as opposed to differentiation from the healthy.

Reported T_2_ and T_2_^*^ values in healthy subjects showed large variation between studies, which could partly be due to the lack of acquisition standardization. In the standardized CMR imaging guideline and protocol published in 2013 [194], T_2_^*^ mapping was only described as a clinical applicable technique to assess cardiac iron deposition and T_2_ mapping was defined as a research-domain technique [194, 195]. T_2_ mapping sequences were stated as optional since there was no standardization yet [194], which led to different acquisition approaches and therefore potentially acquisition related variation in T_2_ values. In 2017, clinical recommendations were released regarding parametric imaging of both T_2_ and T_2_^*^ mapping and defined standardized data acquisition and analysis [22]. They stated that local healthy T_2_ and T_2_^*^ values should be determined in order to clinically use these quantitative techniques, which is now confirmed by this meta-analysis considering the wide variation of healthy T_2_ and T_2_^*^ values (Figs. 2, 3, 5 and 6). The use of normal scan results of clinically referred patients could be used to determine reference values, however this is not recommended due to referral bias. Age- and gender-matching of the control group is necessary [22], since both are known to influence T_2_ and T_2_^*^ values [30]. Furthermore, the clinical recommendations also stated specific imaging protocols, technical requirements of sequences and image planning for T_2_ and T_2_^*^ mapping, which should reduce variability in image acquisition from then onward [22]. This meta-analysis includes multiple studies that were published prior to this guideline and showed the heterogeneity to be significantly influenced by the sequence based covariates, which has previously already been concluded from a direct comparison between sequences [196]. This analysis also showed the variation between CMR vendors with on 1.5 T healthy control T_2_ values of 54.9 ± 3.3 ms at Philips (*n* = 13 studies) and 50.0 ± 2.5 ms at Siemens (*n* = 22) and T_2_^*^ values of 34.1 ± 6.5 ms at Philips (*n* = 5), 30.8 ± 4.5 ms at Siemens (*n* = 3) and 55.0 ± 13.0 ms at General Eletric (GE) (*n* = 1), and on 3 T healthy control T_2_ values of 44.7 ± 5.8 ms at Philips (*n* = 6) and 48.0 ± 3.0 ms at Siemens (*n* = 5), and T_2_^*^ values of 23.9 ± 4.7 at Philips (*n* = 2), 21.0 ± 4.8 ms at Siemens (*n* = 1) and 21.0 ± 6.4 ms at GE (*n* = 1). These differences in vendor and field strength should be kept in mind when T_2_ and T_2_^*^ values are used within a clinical protocol.

In addition to the clinical guideline on T_2_ and T_2_^*^ acquisitions [22], following the recommendations in image analysis could reduce the non-physiological variation of T_2_ and T_2_^*^ values. The clinical recommendations on acquisition and ROI placement are described specifically per disease [22], and this meta-analysis confirmed the different approaches in analysis. In general the ROI should be placed outside positive LGE myocardium areas and include non-fibrous myocardium [22]. T_2_ values measured in positive LGE myocardium should therefore be interpreted cautiously. Analysis of T_2_ in diffuse diseases, such as HCM and DCM, were mostly performed based on one or three short axis (SAx) slices using global assessment [162, 164–169], as recommended [22]. In patchy diseases, such as amyloidosis and Anderson-Fabry disease, the recommendations state that the T_2_ analysis should also include a single 3 chamber or 4 chamber view acquisition additionally to basal and mid-ventricular SAx slices [22]. Only one study actually followed these recommendations [158], while for the other cardiac patchy disease studies one or more recommended slices were not included [155–157]. In focal diseases, such as MI and myocarditis, the ROI differs between patients because the location of the abnormality is different, and therefore the guideline recommends multiple SAx acquisition to cover the whole myocardium and to place the ROI in visually abnormal myocardium [22]. Most included studies in this meta-analysis therefore acquired multiple SAx slices [51, 54–56, 61, 63, 65], however some studies acquired only one [60] or three [49] SAx slices at the level of the infarcted area, which is more prone to missing the infarct core. In the studies with myocarditis patients mapping acquisition was generally also performed over multiple SAx covering the whole myocardium [38, 171–173, 175–180, 182, 183, 185], however in some studies the T_2_ values were only acquired from a LGE hyperintense based ROI [25, 174, 181, 184, 187]. Also, studies including MI, often distinguish between the infarct region or core and use remote myocardium as the healthy control tissue. In these studies the ROI placement was generally based on LGE hyperintense regions [26, 41, 49, 51, 57, 58, 60–63, 65, 67, 68], 2SD change of T_2_ signal intensity [40, 43, 54, 56, 59, 60] or T_2_^*^ values [41, 43, 56]. This meta-analysis showed that ROI placement significantly influences the T_2_ and T_2_^*^ outcome and the separate analysis showed the infarct zone to have a larger T_2_ difference with controls than the infarct core, while the infarct core showed a larger T_2_^*^ difference with controls than the infarct zone. Lastly, for studies including iron overload patients most T_2_^*^ measurements were performed in the intraventricular septum for reproducibility, because the lateral wall often contains dephasing artefacts. Nevertheless, some studies reported an average of the mid-ventricular SAx slice [87, 115, 119, 134] or the entire myocardium [106, 125, 127–132], which especially on 3 T [127] could lead to some unrealistic T_2_^*^ values due to aforementioned artefacts.

In this meta-analysis including MI patients other covariates aside from the ROI placement had a significant effect on T_2_ and T_2_^*^ mapping outcomes. These covariates included the use of remote myocardium as control values instead of healthy controls, the timing of CMR acquisition after reperfusion, and the sequence that was used. The first covariate that included the use of remote myocardium as control, showed that remote myocardium is physiologically different from healthy tissue and therefore is not an appropriate control tissue [197, 198]. Followed by the second covariate for timing of the CMR imaging after PCI, for which histologically is verified in swine that edema and haemorrhage formation peaks in the acute phase 2 h and 7 days post-PCI [199] . These peaks were also detected in the acquired T_2_ values in humans at the same day and at 10 days post-PCI, compared to 3 days post-PCI [43]. These results were contradicted by another study [64] that reported higher T_2_ values at 3 days post-PCI compared to the same day or at 7 days post-PCI. The third covariate showed that the use of a spin-echo based sequence provides larger differences between MI patients and controls, than the gradient-echo-spin-echo or T_2_-prepared balanced steady-state free procession sequences, while the latter two are currently recommended in the general guideline [22]. Lastly due to the remaining high heterogeneity of the MI meta-analysis other covariates are expected to influence the T_2_ and T_2_^*^ mapping outcomes in addition to the ones identified here.

In this meta-analysis including heart transplant patients the main distinct covariate was the rejection status of the transplanted heart. Acute cellular rejection is characterized by infiltration of inflammatory cells accompanied with edema resulting in higher T_2_ values [22, 200], which was also reported in most included studies [22, 27, 71–73, 75, 76, 200]. Nevertheless, patients with negative biopsies also showed higher T_2_ values than controls [69, 71, 75], suggesting that the higher T_2_ values in heart transplant patients may also be related to the inflammatory changes from the transplantation process. The exploratory meta-analysis, however, indicated that positive rejection was a significant covariate to result in larger differences of T_2_ values between heart transplant patients and healthy controls [27, 72, 73, 77], and therefore further research is needed to investigate the clinical applicability of T_2_ mapping for early detection of heart transplant rejection.

In this meta-analysis all transfusion-dependent diseases leading to iron overload were evaluated in one group including thalassemia, sickle cell disease and anaemias [201]. The overall average T_2_^*^ value for iron overload patients was 27.2 ± 13.7 ms, which was above the established iron overload cut-off (T_2_^*^ < 20 ms) [195]. This could be due to the fact that most studies reported T_2_^*^ values without distinguishing between cardiac or non-cardiac iron overload involvement. Some studies provided T_2_^*^ values of cardiac involved patients using < 20 ms as a clinical cut-off [22]. Consequently, the mean T_2_^*^ value of these cardiac involved patients was only 11.8 ± 3.7 ms, which was significantly lower than the controls. The type of controls should ideally only include healthy volunteers, however in some studies also non-cardiac involved iron overload patients were used as controls. The T_2_^*^ value from real healthy volunteers of 32.4 ± 5.6 ms [79, 81, 85, 88, 93, 107, 118, 133] was lower than the 35.7 ± 6.4 ms from non-cardiac iron overload patients [95, 96, 104, 113, 114, 124, 127, 132], and therefore the accuracy of the T_2_^*^ < 20 ms cut-off to establish cardiac involvement could be challenged. The current recommendation advises to perform T_2_^*^ mapping on 1.5 T, since higher field strengths show more susceptibility artefacts [22]. Nonetheless, two studies [81, 88] were performed at 3 T as well as 1.5 T including patients and controls, in which ROI placement was performed at the mid-ventricular septum to avoid susceptibility artefacts [22]. As expected, these studies showed a larger SMD between healthy controls and iron overload patients at 3 T compared to 1.5 T (SMD of − 0.27 and − 0.16), since the transverse relativity of paramagnetic substrates increases with field strength [202]. These last findings show that iron overload evaluation on 3 T seems to be a trade-off between increased risk on artefacts and a higher iron sensitivity.

Furthermore, T_2_ mapping was expected to be sensitive for iron overload [22], however this was not unequivocally confirmed by this meta-analysis (SMD = − 0.54, *P* = 0.30). One study performed on 1.5 T and 3 T showed no statistically significant T_2_ changes in iron overload patients [81], while others did show clear changes in T_2_ values [82, 93, 101]. In this first study only 6% of their patients had cardiac involvement, which might explain the lack of change in T_2_. The other studies showed a high correlation between T_2_ and T_2_^*^ changes and significantly lower T_2_ values in patients with cardiac involved iron overload compared to healthy controls suggesting that T_2_ to could indeed be sensitive to iron overload [82, 93, 101]. More research is needed to validate this conclusion.

In Anderson-Fabry disease only patients with LVH showed significantly higher T_2_ values compared to healthy controls [158]. Previous research showed that native T_1_ mapping is the most sensitive CMR parameter in Anderson-Fabry disease and that Anderson-Fabry disease patients showed lower T_1_ values than controls regardless of LV function and morphology, and therefore T_1_ mapping is also sensitive to distinguish between controls and Anderson-Fabry disease patients without LVH [203]. One study, which was not included within this meta-analysis because it was published previous to our search period, also reported higher T_2_ values in Anderson-Fabry disease patients compared to both HCM patients and healthy controls, suggesting that T_2_ mapping is also a sensitive CMR marker to early assess cardiac involvement in Anderson-Fabry disease patients without LVH [204].

The higher T_2_ values in DCM patients found in this meta-analysis confirmed the immunohistologal evidence of chronic myocardial inflammation for this disease [205]. Studies reporting T_2_ values of DCM subgroups seemed contradicting, since one study [166] showed higher T_2_ values in severe DCM compared to mild DCM (*P* <  0.05), while another [167], though not significant, showed lower T_2_ values in severe DCM compared to mild DCM. Nevertheless, overall higher T_2_ values in DCM patients was confirmed by this meta-analysis.

This meta-analysis including studies with myocarditis patients confirmed the expected higher T_2_ values in the acute phase. All studies reported significantly higher T_2_ values except for one study that showed non-significantly higher T_2_ values in the acute phase compared to healthy controls, with 65.3 ± 45.4 ms and 53.7 ± 31.0 ms, respectively, which was mainly due to the broad SD of both groups [184]. Aside from the higher T_2_ values in the acute phase, a follow-up study showed that 3 and 12 months after symptom onset the T_2_ values returned to normal [174]. Another follow-up study confirmed these normal T_2_ values at 189 days after symptom onset, and also showed that after 40 days the T_2_ values were still significantly higher compared to healthy controls, with 52.4 ± 1.0 ms and 50.4 ± 2.3 ms, respectively [185]. These follow-up studies suggest that T_2_ mapping in myocarditis is most valuable in the acute phase in addition to the Lake Louise criteria that include histology and CMR with T_1_- and T_2_-weighted imaging.

The single study that reported T_2_ values from HCM patients and controls showed significantly higher T_2_ values in patients [158]. Two studies compared the T_2_^*^ values from HCM patients with healthy controls, however their results were contradicting. One study at 1.5 T reported significantly lower T_2_^*^ values in HCM patients compared to controls with 26.2 ± 4.6 ms and 31.3 ± 4.3 ms, respectively [159], whereas the other study at 3 T reported no significant difference with 22.3 ± 4.1 ms and 21.0 ± 6.4 ms, respectively [160]. Since early treatment is key for HCM patients, it is important to be able to distinguish LVH changes due to either HCM or to hypertension. Differentiating between HCM and hypertension related LVH using only parametric imaging is not possible, as this differentiation depends on multiple clinical factors [13]. Nevertheless one study reported on hypertension patients and showed lower T_2_^*^ values at 3 T for both hypertension patients with LVH (23.8 ± 3.1 ms) and without LVH (28.6 ± 4.2 ms) compared to healthy controls (30.8 ± 2.7 ms) [50]. Based on these limited available studies no conclusion can be drawn on the clinical relevance of T_2_ and T_2_^*^ mapping. More research could enable to determine the clinical applicability of these mapping techniques, while T_1_ mapping has already shown to be promising in distinguishing hypertension related LVH and HCM [21, 206]. Furthermore, as the incidence of cardiomyopathies is related to obesity and T2DM [8] it is important to determine whether these high cardiovascular risk factors cause myocardial tissue adaptation and if these are distinguishable with quantitative techniques. Unfortunately, no T_2_ and T_2_^*^ mapping of these risk populations is yet, and therefore we have to rely on the values of cardiac diseases without considering these risk factors.

## Conclusion

This meta-analysis showed that T_2_ and T_2_^*^ values of both patients and healthy controls demonstrate variation between studies related to differences in population demographics, CMR vendor, acquisition methods and analysis approach. This variation limits comparison between centers and therefore each center requires local T_2_ and T_2_^*^ reference values to distinguish affected myocardium in cardiomyopathies from healthy myocardium. To this end reference values should be obtained in, preferably matched, healthy controls using the same CMR acquisition method as in patient care. Although similarities of changes in T_2_ and T_2_^*^ values between cardiac diseases limits direct differentiation, this paper provides T_2_ and T_2_^*^ mapping data which, together with other CMR parameters such as T_1_ mapping, ECV and LGE, can help to differentiate between cardiac disease entities.

## Supplementary information

**Additional file 1.**

**Additional file 2: Figure 1.** Weighted mean T_2_^*^ values and weighted standard deviations (SD) of the sub-analysis in patients with myocardial infarction and iron overload measured at 1.5 T (A) and 3 T (B). In myocardial infarction, T_2_^*^ values of remote myocardium (r) (grey square), infarct core (c) (black square) and infarct zone (z) (black triangle) are presented. In iron overload, the T_2_^*^ value of iron overload patients (p) with cardiac involvement is presented. The number of included measurements for each population is reported above the graph. *MI* myocardial infarction, *IO* iron overload. **Figure 2.** Weighted mean T_2_ values and weighted standard deviations (SD) of the sub-analysis in patients with myocardial infarction, heart transplantation and myocarditis measured at 1.5 T (A) and 3 T (B). In myocardial infarction, T_2_ values of remote myocardium (r) (grey square), infarct core (c) (black square) and infarct zone (z) (black triangle) are presented. In heart transplantation, T_2_ values of heart transplant recipients with negative rejection (n) (grey square) and positive rejection (p) (black square) are presented. In myocarditis, T_2_ values of populations scanned in the non-acute phase (n) (grey square) and in the acute phase (a) (black square) are presented. The number of included subjects for each population is reported above the graph. MI *myocardial infarction,* Trans *heart transplantation,* MC *myocarditis.*

## Data Availability

The data generated to reach the conclusions of this meta-analysis are available from the corresponding author on reasonable request.

## Abbreviations

CMR: Cardiovascular magnetic resonance
CI: Confidence interval
DCM: Dilated cardiomyopathy
ECG: Electrocardiogram
GE: General Electric
HCM: Hypertrophic cardiomyopathy
HF: Heart failure
IO: Iron overload
LGE: Late gadolinium enhancement
LVEF: Left ventricular ejection fraction
LVH: Left ventricular hypertrophy
MI: Myocardial infarction
NICM: Non-ischemic cardiomyopathy
NOS: Newcastle-Ottawa quality assessment scale
NSTEMI: Non-ST elevation myocardial infarction
PCI: Percutaneous coronary intervention
PRISMA: Preferred Reporting Items for Systematic Reviews and Meta-Analyses
ROI: Region-of-interest
SAx: Short axis
SCMR: Society for Cardiovascular Magnetic Resonance
SD: Standard deviation
SLE: Systemic lupus erythematosus
SMD: Standardized mean difference
STEMI: ST-elevation myocardial infarction
T2DM: Type 2 diabetes mellitus
