# Correction to: Cardiovascular magnetic resonance native T_2_ and T_2_^*^ quantitative values for cardiomyopathies and heart transplantations: a systematic review and meta-analysis

**DOI:** 10.1186/s12968-020-00646-8

**Published:** 2020-06-15

**Authors:** G. J. H. Snel, M. van den Boomen, L. M. Hernandez, C. T. Nguyen, D. E. Sosnovik, B. K. Velthuis, R. H. J. A. Slart, R. J. H. Borra, N. H. J. Prakken

**Affiliations:** 1 Department of Radiology, University Medical Center Groningen, University of Groningen, Hanzeplein 1, 9713 GZ Groningen, The Netherlands; 2 Department of Radiology, Athinoula A. Martinos Center for Biomedical Imaging, Massachusetts General Hospital, Harvard Medical School, 149 13th Street, Charlestown, MA 02129 USA; 3 Cardiovascular Research Center, Massachusetts General Hospital, Harvard Medical School, 149 13th Street, Charlestown, MA 02129 USA; 4 Division of Health Sciences and Technology, Harvard-MIT, 7 Massachusetts Avenue, Cambridge, MA 02139 USA; 5 Department of Radiology, University Medical Center Utrecht, Heidelberglaan 100, 3584 CX Utrecht, The Netherlands; 6 Department of Nuclear Medicine and Molecular Imaging, University Medical Center Groningen, University of Groningen, Hanzeplein 1, 9713 GZ Groningen, The Netherlands; 7 Department of Biomedical Photonic Imaging, University of Twente, Dienstweg 1, 7522 ND Enschede, The Netherlands

**Correction to: J Cardiovasc Magn Reson (2020) 22: 34**

**https://doi.org/10.1186/s12968-020-00627-x**

In the original publication [1] of this article there was a typesetting error in Figs. 5 and 6. The captions were correct, but the figures were swapped.

**Fig. 5:**
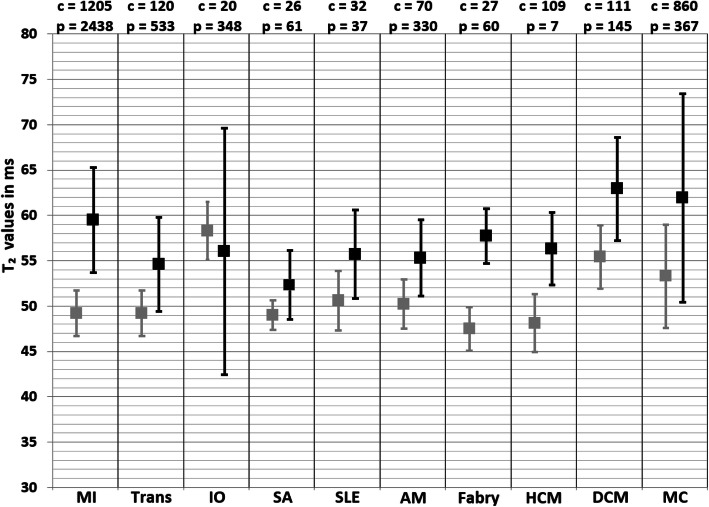
Weighted mean T2 values and weighted standard deviations (SD) of all included papers reporting T2 values of both patients (black squares) and controls (grey squares) measured at 1.5 T. The number of included patient (p) and control (c) measurements for each population is reported above the graph. MI myocardial infarction, Trans heart transplant, IO iron overload, SA sarcoidosis, SLE systemic lupus erythematosus, AM amyloidosis, HCM hypertrophic cardiomyopathy, DCM dilated cardiomyopathy, MC myocarditis

**Fig. 6:**
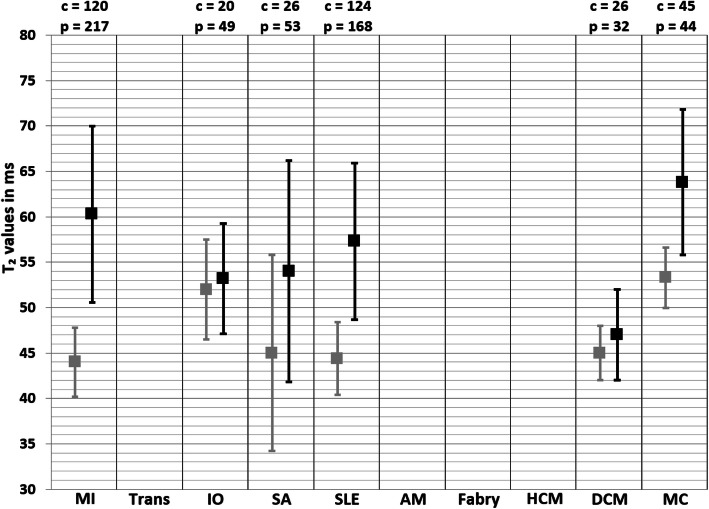
Weighted mean T2 values and weighted standard deviations (SD) of all included papers reporting T2 values of both patients (black squares) and controls (grey squares) measured at 3 T. The number of included patient (p) and control (c) measurements for each population is reported above the graph. MI myocardial infarction, Trans heart transplant, IO iron overload, SA sarcoidosis, SLE systemic lupus erythematosus, AM amyloidosis, HCM hypertrophic cardiomyopathy, DCM dilated cardiomyopathy, MC myocarditis

In this correction article the corrected figures are published. The original article has been updated.

The publisher apologizes to the authors & readers for the inconvenience.
