# Supplementary material for: Cardiovascular magnetic resonance native T2 and T2* quantitative values for cardiomyopathies and heart transplantations: a systematic review and meta-analysis
Source: J Cardiovasc Magn Reson. 2020 May 11;22:34. doi: 10.1186/s12968-020-00627-x (PMC7212597; doi:10.1186/s12968-020-00627-x)
Supplement: Supplementary file 1 — Additional file 1. [file 12968_2020_627_MOESM1_ESM.docx]

**PubMed search term**

(((T2 [tiab] OR “T 2”[tiab] OR t2star[tiab]) **AND** (mapping [tiab]OR maps [tiab] OR relaxation [tiab] OR value [tiab] OR values[tiab] OR time[tiab] OR times[tiab])) **AND** (“Magnetic Resonance Imaging”[Mesh] OR “Magnetic Resonance” [tiab] OR MRI[tiab] OR “cardiac MR” [tiab] OR CMR[tiab] OR “MR imaging”[tiab] OR MR[tiab]))

**AND** ((“heart diseases” [Mesh] OR “heart disease” [tiab] OR myocard* [tiab] OR “heart failure”[Mesh] OR “heart failure”[tiab] OR “cardiac injury”[tiab]) OR ((“Heart” [Mesh] OR Heart [tiab] OR cardiac[tiab]) **AND** (“Sarcoidosis” [Mesh] OR Sarcoidosis [tiab]OR Sarcoidoses [tiab] OR “Besnier Boeck Schaumann” [tiab] OR “Amyloidosis” [Mesh] OR Amyloidosis [tiab] OR amyloidoses [tiab] OR “Churg-Strauss Syndrome"[Mesh] OR "Churg-Strauss*" [tiab] OR “Fabry Disease”[Mesh] OR “Anderson Fabry”[tiab] OR “Fabry Disease”[tiab] OR “Anderson-Fabry”[tiab] OR “Cardiomyopathies”[Mesh] OR cardiomyopathy [tiab] OR cardiomyopathies [tiab] OR “Myocarditis” [Mesh] OR myocarditis[tiab] OR “Iron Overload” [Mesh] OR “Iron overload”[tiab] OR "Hemochromatosis"[Mesh] OR hemochromatosis [tiab] OR haemochromatosis [tiab] OR Hemosiderosis [tiab] OR Haemosiderosis [tiab] OR “Coronary artery disease”[ Mesh] OR “Coronary artery disease”[tiab]OR “Coronary Atherosclerosis”[tiab] OR “Transplantation”[Mesh] OR Transplantation [tiab])) OR (“Edema, Cardiac”[Mesh] OR “cardiac edema”[tiab] OR “cardiac oedema”[tiab]) OR ((Myocardial[tiab] OR Cardiac[tiab]) AND (inflammation [tiab] OR infiltration [tiab] OR edema[tiab] OR necrosis[tiab] OR “hemorrhage”[Mesh] OR hemorrhage[tiab] OR haemorrhage [tiab] OR “Connective Tissue Diseases”[Mesh] OR “Connective Tissue”[tiab])) **OR (**(Myocardial[tiab] OR Cardiac[tiab] OR “Heart” [Mesh] OR Heart [tiab]) **AND** ("Obesity"[Mesh] OR "Overweight"[Mesh] OR “body weight”[tiab] OR “body mass index”[tiab] OR obese [tiab] OR obesity[tiab] OR overweight [tiab] OR "Diabetes Mellitus"[Mesh] OR diabetes [tiab] OR diabetic[tiab] OR "Hypertension"[Mesh] OR “blood pressure"[Mesh] OR hypertension [tiab] OR hypertensive[tiab] OR "high blood pressure"[tiab])))

**NOT** ((“Animals”[Mesh] NOT “Humans”[Mesh]) OR pigs[tiab] OR pig[tiab] OR swine[tiab] OR mice[tiab] OR rats[tiab] OR rabbit[tiab] OR rabbits[tiab] OR “child”[mesh] OR children[tiab] OR Case Reports[ptyp] OR cancer[tiab] OR chemotherapy[tiab] OR “Neoplasms”[Mesh] OR “cartilage” [Mesh] OR cartilage[tiab] OR “stroke”[Mesh] OR “Brain Infarction”[Mesh])

**AND** ("2011/01/01"[PDat] : "2019/08/31"[PDat])

Final search date: 4 September 2019
