# Supplementary figures and images for: Cardiovascular magnetic resonance native T2 and T2* quantitative values for cardiomyopathies and heart transplantations: a systematic review and meta-analysis
Source: J Cardiovasc Magn Reson. 2020 May 11;22:34. doi: 10.1186/s12968-020-00627-x (PMC7212597; doi:10.1186/s12968-020-00627-x)

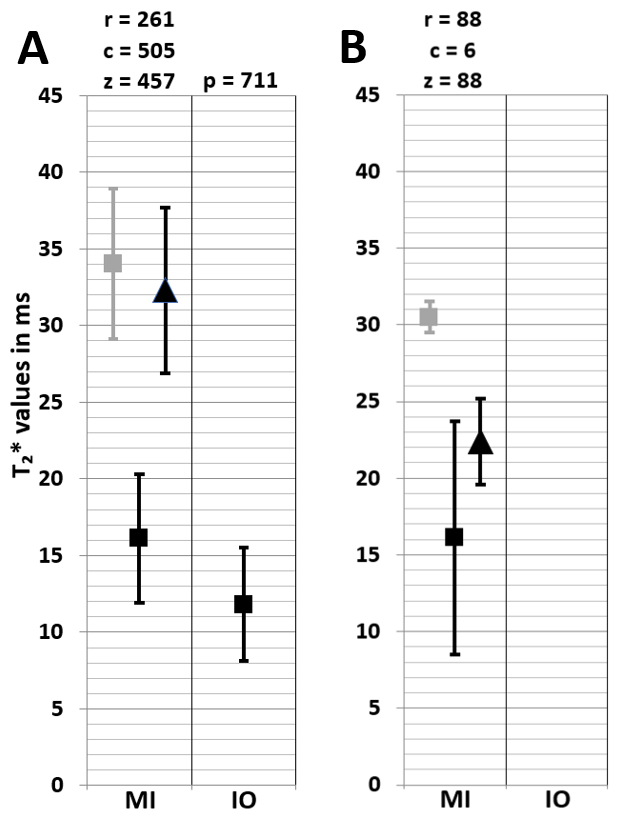


Fig. 1


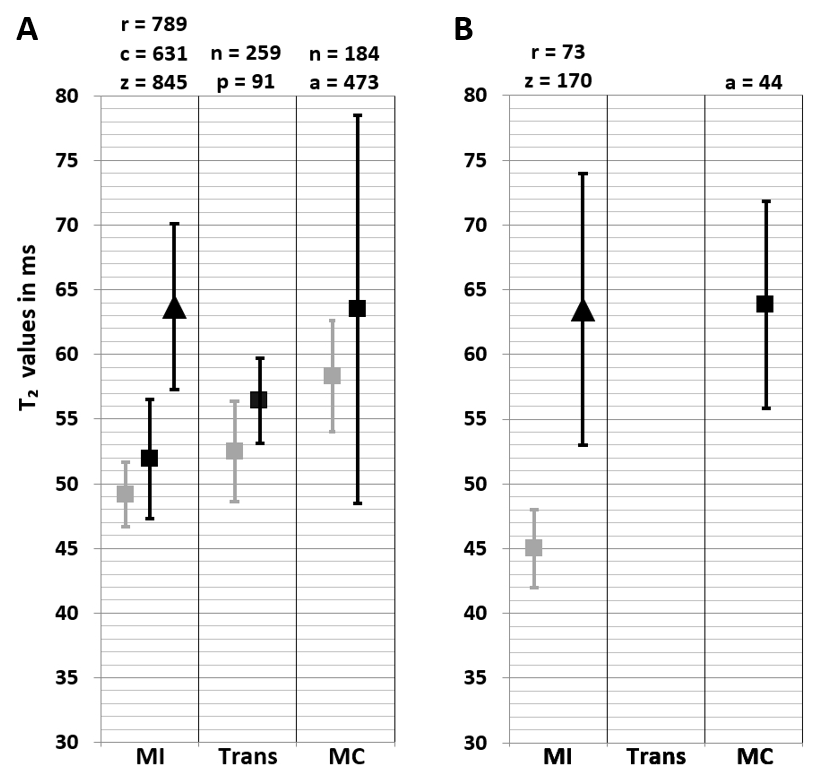


Fig. 2

Supplement: Supplementary file 2 — Additional file 2: Figure 1. Weighted mean T2* values and weighted standard deviations (SD) of the sub-analysis in patients with myocardial infarction and iron overload measured at 1.5 T (A) and 3 T (B). In myocardial infarction, T2* values of remote myocardium (r) (grey square), infarct core (c) (black square) and infarct zone (z) (black triangle) are presented. In iron overload, the T2* value of iron overload patients (p) with cardiac involvement is presented. The number of included measurements for each population is reported above the graph. MI myocardial infarction, IO iron overload. Figure 2. Weighted mean T2 values and weighted standard deviations (SD) of the sub-analysis in patients with myocardial infarction, heart transplantation and myocarditis measured at 1.5 T (A) and 3 T (B). In myocardial infarction, T2 values of remote myocardium (r) (grey square), infarct core (c) (black square) and infarct zone (z) (black triangle) are presented. In heart transplantation, T2 values of heart transplant recipients with negative rejection (n) (grey square) and positive rejection (p) (black square) are presented. In myocarditis, T2 values of populations scanned in the non-acute phase (n) (grey square) and in the acute phase (a) (black square) are presented. The number of included subjects for each population is reported above the graph. MI myocardial infarction, Trans heart transplantation, MC myocarditis. [file 12968_2020_627_MOESM2_ESM.docx]
